# Electrosprayed PLGA Nanoparticles for Dual Drug Delivery: Design, Optimization and Applications

**DOI:** 10.3390/polym18091133

**Published:** 2026-05-05

**Authors:** Bahareh Azimi, Fatemeh Ahmadpoor, Alessia Tozzi, Afsaneh Shahraki, Homa Maleki, Pier Francesco Ferrari, Serena Danti

**Affiliations:** 1 Department of Civil and Industrial Engineering, University of Pisa, largo Lucio Lazzarino 2, 56122 Pisa, Italy; a.tozzi7@studenti.unipi.it (A.T.);; 2 National Interuniversity Consortium for Materials Science and Technology (INSTM), Via Giusti 9, 50121 Florence, Italy; 3 Department of Civil, Chemical and Environmental Engineering, University of Genoa, Via Opera Pia 15, 16145 Genoa, Italy; 4 Faculty of Arts, University of Birjand, Birjand 9717434765, Iran; 5 Research Center for Biologically Inspired Engineering in Vascular Medicine and Longevity, University of Genoa, Via Montallegro 1, 16145 Genoa, Italy; 6 IRCCS Azienda Ospedaliera Metropolitana, Largo Rosanna Benzi 10, 16132 Genoa, Italy

**Keywords:** electrospraying, PLGA nanoparticle, drug release kinetics, mathematical modelling, targeted drug delivery, nanoparticle detachment

## Abstract

Poly(lactic-*co*-glycolic acid) (PLGA) is a biodegradable biopolymer widely used in advanced drug delivery systems (DDSs) due to its biocompatibility, controllable degradation behavior, and tunable physicochemical properties. Its degradation into naturally metabolized lactic and glycolic acids makes PLGA particularly attractive for biomedical applications, positioning PLGA nanoparticles as versatile carriers that bridge material design and therapeutic delivery. In this context, electrospray (electrohydrodynamic atomization) has emerged as an innovative and scalable processing technique that enables precise control over nanoparticle size, morphology, and internal structure under mild conditions, which is particularly suitable for engineering biopolymer-based DDSs. This review provides a comprehensive overview of electrospray-fabricated PLGA nanoparticles, with emphasis on the relationship between processing conditions, polymer structure, and functional performance. The fundamental mechanisms governing drug release, including diffusion, polymer degradation, and their combined effects, are discussed in relation to PLGA properties. The influence of electrospray parameters on nanoparticle formation, morphology, and internal architecture is analyzed, highlighting how process–structure–property relationships can be tailored to achieve specific release profiles. Structural design strategies, including single-matrix, core–shell, and surface-functionalized nanoparticles, are further examined as approaches to enable controlled and sequential dual-DDSs. In addition, emerging modeling and computational approaches are briefly discussed as complementary tools for understanding and optimizing nanoparticle behavior. Challenges and technical problems, such as substrates for nanoparticle detachment, are discussed.

## 1. Introduction

Polymeric drug delivery systems encompass a wide range of material platforms, including nanoparticles, micelles, dendrimers, hydrogels, and polymer–drug conjugates, each designed to address specific therapeutic and structural requirements. These systems are fabricated from both natural polymers (e.g., chitosan, alginate, gelatin) and synthetic polymers such as poly(lactic acid) (PLA), polycaprolactone (PCL), polyethylene glycol (PEG), and poly(lactic-*co*-glycolic acid) (PLGA), which offer tunable physicochemical properties and controlled degradation behavior. The selection of polymer type and delivery platform plays a critical role in determining drug loading capacity, release kinetics, biocompatibility, and targeting capability [1].

In addition to nanoparticle-based fabrication methods, several advanced processing platforms have been explored for polymer-based drug delivery systems, each offering distinct structural and functional advantages. Electrospinning enables the production of nanofibrous scaffolds that mimic the extracellular matrix, providing structural support and facilitating localized and sustained drug release [2]. In contrast, additive manufacturing (3D printing) allows the fabrication of polymeric devices with precise, patient-specific geometries, enabling spatial control over drug distribution and release behavior [3]. Conventional molding techniques also remain relevant for producing implantable systems with well-defined shapes and mechanical properties, as demonstrated by perivascular wraps designed for localized therapeutic delivery and vascular remodeling [4,5]. Compared to these approaches, electrospraying is particularly suited for generating discrete micro- and nanoparticles with controlled size and morphology, making it highly effective for systemic and injectable drug delivery applications. Therefore, the selection of fabrication technique must be aligned with the intended therapeutic application, required structural features, and delivery route.

**Figure 1:**
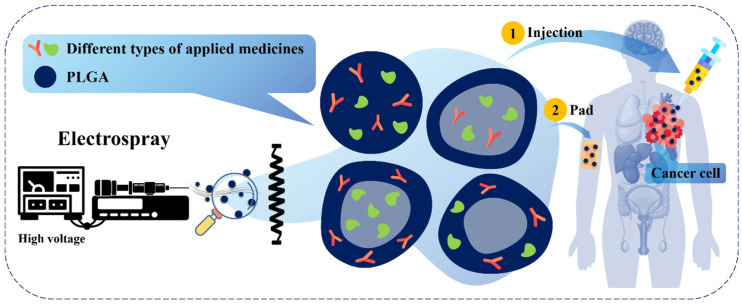
Schematic illustration of electrospray-based fabrication of PLGA nanoparticles for dual-drug delivery. A polymer solution is atomized under a high-voltage electric field to form nanoparticles with different drug distribution architectures, including matrix-type particles and core–shell structures, where drugs are either uniformly dispersed or spatially separated (e.g., drug-loaded core with a polymer or drug-containing shell). For in vivo applications, nanoparticles can be administered via systemic injection or localized delivery (e.g., transdermal pad), enabling controlled drug release at the target site (created by the authors). Different colors and shapes represent different types of applied medicines, as indicated in the figure.

Among these systems, PLGA has gained particular attention due to its excellent biocompatibility, biodegradability, and regulatory approval for clinical use. The degradation of PLGA into lactic and glycolic acids, which are naturally metabolized in the body, makes it especially suitable for biomedical applications. In addition, the physicochemical properties of PLGA, including molecular weight, copolymer ratio, and end-group functionality, can be precisely tuned to achieve controlled and sustained drug release profiles [6]. Over the past decades, PLGA-based nanoparticles have been widely investigated for the delivery of a broad range of therapeutic agents, including small-molecule drugs, proteins, peptides, and nucleic acids [7]. These systems have demonstrated significant potential in cancer therapy and immunotherapy, where precise control over drug release kinetics and targeted delivery is critical for therapeutic success. In particular, combination therapy involving multiple agents has attracted increasing interest, as it enables synergistic effects, reduces drug resistance, and allows simultaneous modulation of multiple biological pathways [8,9,10]. Conventional fabrication methods for PLGA nanoparticles, such as emulsion–solvent evaporation and nanoprecipitation, have been extensively employed; however, these techniques often involve complex processing steps, surfactant use, and limited control over particle size distribution and morphology [11,12]. In this context, electrospray has emerged as a promising alternative fabrication technique. By utilizing electrostatic forces to generate fine droplets, electrospray enables the production of monodisperse nanoparticles with tunable size, morphology, and internal structure [13]. Compared with conventional methods such as high-shear emulsification or spray drying, electrospray can operate under relatively mild processing conditions, avoiding intense mechanical stresses and elevated temperatures. However, the process is not entirely benign; exposure to strong electric fields, shear forces at the needle tip, and organic solvent interfaces may still induce structural changes or the denaturation of sensitive biomolecules, particularly proteins and cells, during atomization. These factors must be carefully controlled through optimization of formulation and processing parameters to preserve bioactivity. These advantages are particularly relevant for the encapsulation of sensitive bioactive agents and the design of advanced drug delivery systems [14]. An overview of the electrospray fabrication process, nanoparticle architectures, and their application in PLGA-based dual-drug delivery is illustrated in Figure 1. Recent research has increasingly focused on the development of electrosprayed PLGA nanoparticles for dual-drug delivery, especially in the context of chemo-immunotherapy [15]. Structural designs such as core–shell architectures and surface-functionalized systems allow spatial and temporal control over drug release, enabling sequential or stimuli-responsive delivery strategies. Despite these advances, several challenges remain, including the co-encapsulation of therapeutics with different physicochemical properties, precise control of release kinetics, and the preservation of biomolecule stability during fabrication [16]. In parallel, mathematical modeling and computational approaches have become essential tools for understanding and optimizing both the electrospray process and drug release behavior [17]. The integration of electrohydrodynamic theory, multiphysics simulations, and data-driven methods such as machine learning provides new opportunities for rational design and process optimization of PLGA-based delivery systems [18]. This review provides a comprehensive overview of electrospray-fabricated PLGA nanoparticles, with a particular focus on dual-drug delivery applications. It first discusses the fundamental properties of PLGA and mechanisms of drug release, followed by an analysis of electrospray fabrication principles and key process parameters. The review then examines dual-drug loading strategies, associated challenges, and the role of stimuli-responsive systems. Finally, it highlights recent advances in mathematical modeling, optimization techniques, and biomedical applications in cancer therapy and immunotherapy, offering insights into future research directions in this rapidly evolving field.

## 2. PLGA Nanoparticles for Drug Delivery

### 2.1. PLGA Properties

Polyesters are a class of polymers characterized by ester bond connections within their carbon framework. Aliphatic polyesters, like polyglycolic acid (PGA), polylactic acid (PLA) [19], PCL [20], and PLGA [21], are among the most extensively studied degradable polymers for biomedical applications.

Among these, PLA, PCL, and PLGA have been widely investigated for drug delivery; however, their distinct physicochemical properties significantly influence their performance in therapeutic systems. PCL is a highly crystalline and hydrophobic polymer characterized by very slow degradation kinetics, often requiring months to years for complete resorption. While this property is advantageous for long-term implantable systems, it limits its applicability in drug delivery scenarios where timely and controlled release is required [20]. In contrast, PLA exhibits moderate degradation behavior but remains relatively hydrophobic and semi-crystalline, which can restrict water penetration and lead to less predictable release profiles, particularly for hydrophilic drugs [19].

As a copolymer of lactic acid and glycolic acid, PLGA offers a key advantage through its tunable degradation kinetics. By adjusting the lactide-to-glycolide (LA:GA) ratio, the degradation rate can be precisely controlled, enabling drug release profiles ranging from days to several months. Furthermore, PLGA degrades into lactic acid and glycolic acid, which are naturally metabolized via physiological pathways, contributing to its excellent biocompatibility and widespread regulatory approval for clinical use [21]. Compared to PLA and PCL, PLGA generally exhibits faster and more controllable hydrolytic degradation, making it particularly suitable for applications requiring sustained and predictable drug release.

From a formulation perspective, PLGA also demonstrates superior versatility in nanoparticle engineering. Its tunable hydrophilicity, composition-dependent amorphous structure, and compatibility with multiple fabrication techniques, including emulsion-based methods, nanoprecipitation, and electrospray, enable efficient encapsulation of both hydrophobic and hydrophilic therapeutic agents. In contrast, the higher crystallinity of PLA and PCL can limit drug diffusion and reduce encapsulation efficiency for certain payloads. In addition, PLGA-based nanoparticles exhibit favorable pharmacokinetic behavior, including controlled degradation, reproducible release kinetics, and well-established safety profiles, which have facilitated their broad use in clinically approved drug delivery systems [21].

PLGA is one of the leading biodegradable polymers used in drug delivery systems and implantable devices due to its excellent biocompatibility and controlled biodegradation for clinical use. PLGA is produced through the ring-opening polymerization of cyclic lactide and glycolide monomers and undergo degradation by hydrolysis of its ester bonds in water [21]. PGA, the simplest linear aliphatic polyester, exhibits high crystallinity (45–55%), which makes it low solubility in most organic solvents. With a glass transition temperature (Tg) of 35–40 °C, PGA show high mechanical properties. However, its low solubility and rapid breakdown into glycolic acid render PGA less suitable for polymeric nanoparticle drug delivery systems. Therefore, copolymers like PLGA have been developed for such applications [22].

#### 2.1.1. Polymer Ratios (PLA/PGA)

PLGA plays a central role in the development of controlled drug delivery systems. The remarkable adaptability and widespread use of PLGA in drug delivery systems are rooted in the fine-tuned balance of its physicochemical properties, which govern its interaction with the biological environment and its functional behavior over time. A deep understanding of these molecular characteristics enables the precise design of PLGA-based formulations and the prediction of their in vivo performance, paving the way for tailored drug release profiles and enhanced therapeutic efficacy. The first critical aspect lies in the molecular architecture of PLGA, which is synthesized from lactic acid existing in two chiral forms (L- and D-lactic acid) and glycolic acid. The relative proportion of these monomers, commonly expressed as the lactide-to-glycolide (LA:GA) ratio, is a primary determinant of the physicochemical behavior of the copolymer. Because lactic acid contains a methyl side group while glycolic acid does not, variations in monomer composition directly influence chain packing, intermolecular interactions, and overall polymer structure [23]. The LA:GA ratio strongly affects the hydrophilic–hydrophobic balance of PLGA. Increasing glycolic acid content enhances polymer hydrophilicity due to the absence of the hydrophobic methyl group, facilitating water penetration into the matrix. In contrast, higher lactic acid content increases hydrophobicity and steric hindrance, reducing water uptake. This compositional control over water diffusion is central to tailoring degradation kinetics and drug release behavior [20]. Crystallinity is also governed by monomer composition. PLGA with an equimolar 50:50 LA:GA ratio is typically amorphous due to the disruption of regular chain packing, whereas increasing lactide content promotes partial crystallinity as a result of more ordered chain alignment. Because crystalline domains are less permeable to water than amorphous regions, higher crystallinity generally slows hydrolytic degradation [24]. As a consequence, the LA:GA ratio provides a practical means of modulating degradation rate. PLGA 50:50 commonly exhibits the fastest degradation due to its amorphous structure and enhanced water uptake. In contrast, formulations with higher lactide content (e.g., 75:25 or 85:15) degrade more slowly because of increased hydrophobicity and reduced chain mobility. Through the adjustment of copolymer composition, the degradation timeframe of PLGA can therefore be tuned from weeks to several months, enabling the rational design of drug delivery systems with controlled release profiles [25,26].

#### 2.1.2. Biodegradability

The biodegradability of PLGA is a defining property that underlies its widespread use in controlled drug delivery systems. PLGA degrades primarily through the non-enzymatic hydrolysis of ester linkages within its backbone. Upon exposure to aqueous environments, water penetrates the polymer matrix and cleaves ester bonds, leading to a progressive reduction in molecular weight before significant mass loss becomes evident [26]. This initial decrease in molecular weight precedes structural disintegration and is characteristic of hydrolytically degradable aliphatic polyesters. PLGA is generally classified as a bulk-eroding polymer. In bulk erosion, water diffuses throughout the polymer matrix faster than ester bonds are cleaved, leading to homogeneous degradation across the entire particle. During the initial phase, molecular weight decreases progressively while the device largely maintains its structural integrity. Once a critical molecular weight threshold is reached, rapid mass loss and structural collapse occur. Although surface erosion may arise under specific formulation conditions, bulk degradation predominates in most PLGA-based systems [6]. The rate and extent of this degradation process are influenced by multiple interrelated parameters, including molecular weight, polymer crystallinity, lactic acid to glycolic acid (LA:GA) ratio and end-caps [27]. These factors collectively govern water diffusion, hydrolytic cleavage kinetics, and the accumulation of acidic degradation products within the matrix. A key feature of PLGA degradation is autocatalysis. As ester bonds are cleaved, acidic oligomers and monomers accumulate within the matrix. In systems where diffusion of these degradation products is restricted, local pH decreases within the polymer interior, further accelerating hydrolytic cleavage. This self-catalyzed process can produce heterogeneous degradation patterns, particularly in larger or densely structured matrices [26]. Device architecture significantly influences the extent of autocatalysis. Smaller particles facilitate more efficient diffusion of acidic byproducts, reducing internal pH gradients and promoting more homogeneous degradation. In contrast, larger or poorly porous systems may experience pronounced core acidification due to limited outward diffusion, leading to accelerated internal degradation relative to the surface. Increased porosity enhances water ingress and oligomer transport, thereby accelerating overall degradation while potentially mitigating localized acid accumulation [28,29,30]. Environmental pH further modulates degradation kinetics. Both acidic and alkaline conditions accelerate ester hydrolysis, whereas near-neutral environments may still exhibit enhanced degradation due to internally generated carboxylic end groups. These pH-dependent effects contribute to variability in degradation behavior under different physiological and experimental conditions [31]. Ultimately, PLGA degradation yields lactic acid and glycolic acid, which are endogenous metabolites eliminated via the tricarboxylic acid cycle as carbon dioxide and water [24]. This metabolic compatibility, combined with predictable hydrolytic degradation, underpins the widespread regulatory acceptance of PLGA for biomedical applications.

#### 2.1.3. Molecular Weight

Molecular weight is a primary determinant of the physicochemical and functional performance of PLGA-based drug delivery systems. As an indicator of polymer chain length, molecular weight directly influences the degradation rate, mechanical strength, structural stability, and drug release duration. In general, higher-molecular-weight PLGA exhibits slower degradation because longer polymer chains require more extensive hydrolytic cleavage before solubilization occurs. Consequently, systems fabricated from high-molecular-weight PLGA typically provide prolonged drug release over extended timeframes, whereas low-molecular-weight polymers degrade more rapidly and often display faster release profiles. Molecular weight also affects the mechanical properties of the polymer matrix. High-molecular-weight PLGA forms mechanically stronger and more structurally stable systems due to increased chain entanglement, while low-molecular-weight polymers yield softer matrices that are more susceptible to deformation and pore expansion under osmotic pressure. These differences can influence drug loading capacity, matrix integrity during degradation, and overall release behavior [6]. Beyond average molecular weight, molecular weight distribution plays a critical role. Polymers with broader molecular weight distributions contain a greater proportion of shorter chains and reactive end groups, which can accelerate degradation and alter release kinetics. Experimental studies have demonstrated that drug release profiles are influenced not only by mean molecular weight but also by distribution breadth, highlighting the importance of precise polymer characterization during formulation design [32]. End-group chemistry further influences degradation. PLGA with free carboxylic acid end groups degrades faster than ester-capped counterparts of similar molecular weight due to increased hydrophilicity and susceptibility to hydrolysis. Controlling end-group functionality therefore provides an additional means to tune degradation rate and drug release [33,34,35]. Collectively, molecular weight, its distribution, and end-group structure define the chain-level architecture of PLGA and represent key design parameters for achieving predictable mechanical behavior and controlled drug release.

#### 2.1.4. Glass Transition Temperature

The glass transition temperature (Tg) marks the shift in a polymer from rigid and glassy to flexible and rubbery. Below Tg, limited chain mobility restricts the free volume and diffusion of water and drugs; above Tg, increased mobility enhances diffusion, permeability, and drug release. For most PLGA copolymers, Tg is near or above physiological temperature (37 °C), meaning that the polymer typically exists in a glassy state under in vivo conditions [20]. In this state, chain mobility is limited and diffusion through the matrix is relatively slow. When environmental or formulation conditions bring the system close to or above Tg, the polymer becomes more flexible, increasing diffusion coefficients and accelerating drug transport. Tg is influenced by copolymer composition and chain architecture. Variations in lactide-to-glycolide ratio alter chain packing and intermolecular interactions, leading to measurable differences in Tg. For example, PLGA with higher lactide content generally exhibits higher Tg values, whereas 50:50 compositions show comparatively lower Tg [36]. Copolymer sequence distribution has also been shown to affect Tg, as increased uniformity in comonomer arrangement can reduce Tg due to altered chain interactions [37]. The relationship between Tg and release behavior is particularly evident in burst release phenomena. When the release medium temperature approaches or exceeds Tg, enhanced chain mobility facilitates rapid diffusion of surface-associated or near-surface drug, often resulting in pronounced initial burst release [38,39]. In contrast, when the system remains below Tg, restricted segmental motion limits diffusion, promoting more gradual and sustained drug release from the polymer matrix. Overall, Tg governs chain mobility and diffusion behavior within PLGA systems and therefore represents a critical parameter in predicting and controlling drug release performance.

#### 2.1.5. Crystallinity

Crystallinity refers to the proportion of ordered, tightly packed chain regions within a polymer relative to amorphous domains. Most biodegradable polyesters, including PLGA, are semicrystalline or predominantly amorphous depending on copolymer composition. The structural distinction between crystalline and amorphous regions plays a critical role in water transport, degradation behavior, and drug release kinetics [40]. Amorphous regions have higher free volume and mobility, enabling water penetration and diffusion, while crystalline regions are dense and less permeable, slowing hydrolysis. Thus, degradation in semicrystalline polyesters begins in amorphous domains and later reaches crystalline ones [26]. In PLGA systems, crystallinity is strongly influenced by monomer composition. Equimolar 50:50 lactide-to-glycolide formulations are generally amorphous, whereas increasing lactide content promotes greater chain ordering and partial crystallinity [41]. Because crystalline domains act as diffusion barriers, higher crystallinity is generally associated with slower water uptake, reduced hydrolysis rates, and diminished drug diffusion. Experimental studies confirm the quantitative impact of crystallinity on transport properties [42]. For example, increasing PLGA crystallinity from 40% to 50% was shown to reduce the diffusion coefficient of lidocaine from 6.95 × 10^−14^ cm^2^/s to 3.84 × 10^−14^ cm^2^/s, resulting in a slower release rate [43]. These findings illustrate the direct relationship between structural ordering and mass transport within the polymer matrix. Overall, crystallinity governs water permeability and molecular diffusion in PLGA systems, thereby modulating both degradation progression and drug release behavior.

### 2.2. Mechanisms of Drug Release from PLGA Systems

#### 2.2.1. Diffusion-Controlled Release

In diffusion-controlled PLGA systems, drug transport is governed by concentration gradients and often follows Fickian behavior, where the cumulative amount of drug released is proportional to the square root of time during the early stage. This regime typically dominates before significant polymer degradation occurs and is frequently associated with the initial burst phase. The magnitude of diffusion-controlled release is strongly influenced by matrix porosity and tortuosity. Increased porosity enhances water penetration and provides interconnected pathways for drug transport, while lower tortuosity reduces the effective diffusion path length. Conversely, highly tortuous or poorly porous matrices impose greater resistance to mass transport, resulting in slower release kinetics [44].

- Diffusion through water-filled pores

In hydrated PLGA systems, water penetrates the matrix and forms aqueous channels or pores through which dissolved drug molecules diffuse. This pathway is especially relevant for hydrophilic drugs, peptides, and proteins that cannot readily partition into the hydrophobic polymer phase. The initial burst release is frequently attributed to rapid diffusion of drug molecules located near the particle surface or within pre-existing pores [45]. The effective diffusion coefficient in such systems depends on multiple structural parameters, including porosity and tortuosity. Higher porosity increases available transport pathways, while lower tortuosity reduces the effective diffusion path length. As a result, porous particles generally exhibit faster release kinetics. For example, porous, sponge-like PLGA microspheres have been shown to release encapsulated progesterone more rapidly than nonporous counterparts due to increased surface area and reduced diffusion resistance, often accompanied by a pronounced burst release [46]. Drug-specific properties also influence diffusion behavior. Molecular size, hydrophilicity, and charge affect solubility within aqueous pores and thus modulate the rate of transport through the hydrated matrix [47].

- Diffusion through the polymer phase

In addition to transport through aqueous pores, small hydrophobic drugs may diffuse directly through the polymer phase. In this case, drug molecules partition into the polymer matrix and migrate via segmental chain mobility. This mechanism is less dependent on pore structure but strongly influenced by the physical state of the polymer [48]. Diffusion increases significantly when the matrix transitions from a glassy to a rubbery state, either above the Tg or due to plasticization by absorbed water. Lower-molecular-weight PLGA, which exhibits greater chain flexibility, generally permits faster diffusion than higher-molecular-weight systems, whereas higher-molecular-weight PLGA maintains a more rigid network that initially restricts drug mobility [49]. In practice, diffusion through water-filled pores and diffusion through the polymer phase may occur simultaneously, with their relative contributions determined by drug properties, polymer structure, and matrix hydration state [44].

#### 2.2.2. Degradation- and Erosion-Controlled Release

In degradable polymer systems, erosion refers to the physical loss of material resulting from polymer chain cleavage. Two principal erosion mechanisms are recognized: surface erosion and bulk erosion. The dominant mechanism depends on the relative rates of water penetration and polymer degradation [50]. PLGA undergoes predominantly bulk erosion, as water rapidly permeates the matrix and the hydrolytic cleavage of ester bonds occurs throughout the entire volume of the polymer. In this mechanism, molecular weight decreases uniformly before significant mass loss becomes apparent. As degradation progresses, cleavage of polymer chains leads to the formation of pores and microcavities within the matrix, increasing matrix permeability and facilitating drug transport [40]. A defining feature of PLGA bulk erosion is autocatalysis. Acidic degradation products accumulate within the polymer interior, particularly in larger or less porous systems where diffusion is limited. The resulting local pH reduction accelerates further ester bond cleavage, producing heterogeneous internal degradation. This process often contributes to an accelerated release phase at later stages, following an initial diffusion-dominated period. In contrast, surface erosion—where degradation proceeds from the exterior inward and mass loss occurs proportionally to surface area—is not typical for PLGA but may be observed under specific conditions. Because PLGA primarily degrades via bulk erosion, release kinetics often reflect a complex interplay between diffusion and degradation, frequently resulting in biphasic or triphasic release profiles [44].

#### 2.2.3. Combined Diffusion–Degradation Behavior

In most PLGA-based systems, drug release is governed by dynamic interplay between diffusion and polymer degradation rather than by a single dominant mechanism. This coupling frequently produces biphasic or triphasic release profiles. An initial burst phase is commonly observed, driven by rapid diffusion of surface-associated or pore-accessible drug. This may be followed by a lag or slow-release phase in which diffusion through a relatively intact polymer matrix predominates while molecular weight progressively decreases. As bulk degradation advances and internal pore formation increases matrix permeability, an accelerated release phase often emerges due to enhanced drug mobility and autocatalytic chain cleavage. In larger or densely structured particles, heterogeneous core degradation can further amplify this late-stage release. The relative magnitude and duration of each phase depend on polymer characteristics, drug distribution, particle size, and matrix architecture, highlighting the coupled nature of transport and degradation processes in PLGA systems [51].

### 2.3. Influence of Nanoparticle Size, Morphology, and Surface Charge on Drug Release

Beyond polymer chemistry and degradation mechanisms, the physical characteristics of PLGA nanoparticles—particularly size, morphology, and surface charge—significantly influence drug release behavior. These parameters modulate water penetration, diffusion pathways, and matrix degradation kinetics, thereby altering release profiles even when polymer composition remains constant [6,52].

#### 2.3.1. Size

Particle size is one of the most influential determinants of drug release kinetics. Smaller nanoparticles possess a higher surface-to-volume ratio, facilitating rapid water ingress and reduced transport distances. As a result, nanosystems typically exhibit faster initial drug release and a more pronounced burst phase compared to larger particles of identical composition. In contrast, larger micro- or nanoparticles present longer diffusion distances and reduced relative surface area, often leading to slower cumulative release and extended release duration.

**Figure 2:**
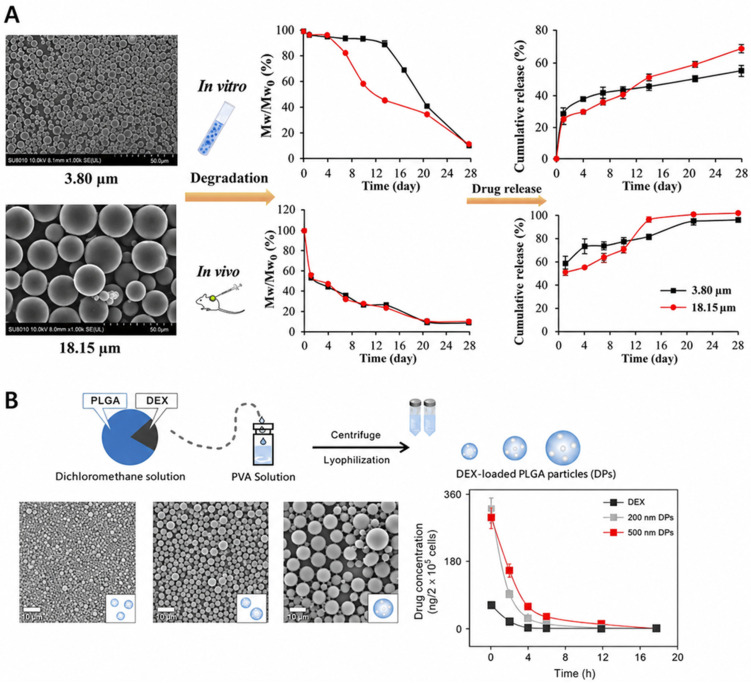
(**A**) Effect of particle size on the degradation and drug release of exenatide-loaded PLGA microspheres. SEM images of 3.80 μm and 18.15 μm microspheres (left). In vitro and in vivo molecular weight reduction (Mw_t_/Mw_0_) and cumulative drug release profiles over 28 days (right). Smaller microspheres showed faster initial release, while in vivo conditions produced a higher burst release for both sizes. Adapted with permission from [53] © 2018 Elsevier B.V. All rights reserved. (**B**) Influence of particle size on encapsulation efficiency (EE) and intracellular drug concentration of dexamethasone-loaded PLGA particles. SEM images of PLGA particles with mean diameters of 200, 500, and 2000 nm (scale bar: 2 μm) (top). EE increases with particle size (bottom left). Intracellular drug concentration profiles (bottom right) demonstrate enhanced cellular uptake and higher initial intracellular levels for 500 nm particles compared to 200 nm particles over time. Reproduced from an open access paper [54] © 2022 Chinese Society of Particuology and Institute of Process Engineering, Chinese Academy of Sciences. Published by Elsevier B.V.

For example, Tang et al. [53] compared the release behavior of exenatide-loaded PLGA microspheres with mean diameters of 3.80 μm and 18.15 μm in vivo. Smaller microspheres exhibited a faster initial release rate due to their higher surface-to-volume ratio and shorter diffusion pathways. However, complete drug release required a longer duration compared to larger particles. Both size groups demonstrated a pronounced burst release in vivo, which was significantly higher than that observed under in vitro conditions. This enhanced in vivo burst was attributed to the steeper concentration gradient and rapid clearance of released drug by surrounding body fluids, which continuously maintained sink conditions and enhanced drug transport (Figure 2A).

Particle size was identified as a critical determinant of the cellular internalization pathway. Fan et al. [54] investigated the size-dependent uptake of PLGA particles (200, 500, and 2000 nm) and their influence on the sustained release of dexamethasone (Figure 2B). Micrometer-sized particles exhibited higher drug-loading capacity compared to submicrometer particles; however, cellular uptake was significantly greater for the smaller particles. Among the nanoscale formulations, 500 nm PLGA particles demonstrated the most favorable sustained-release profile, balancing efficient internalization with controlled drug release.

Size also affects degradation heterogeneity. In larger particles, the diffusion of acidic degradation products may be limited, promoting internal autocatalysis and potentially contributing to an accelerated release phase at later stages. Conversely, smaller particles allow more efficient outward diffusion of oligomers, often resulting in more homogeneous degradation and more predictable release behavior [55].

#### 2.3.2. Morphology

**Figure 3:**
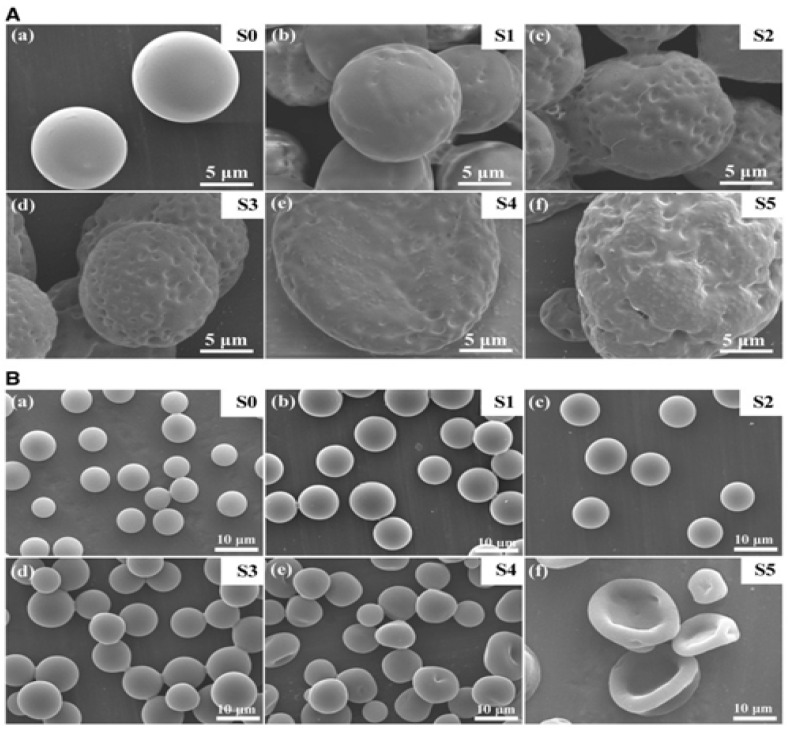
Representative morphologies of BSA-loaded PLGA microspheres prepared at varying aqueous-to-organic phase volume ratios (V_w_/V_o_): 0 (a, S0), 5 (b, S1), 10 (c, S2), 20 (d, S3), 50 (e, S4), and 100 μL/mL (f, S5). Microspheres were fabricated using BSA concentrations of 0.001 g/mL (**A**) and 0.4 g/mL (**B**). Reproduced from an Open Access [58] distributed under the terms of the Creative Commons CC BY 4.0.

Particle morphology further modulates release kinetics by influencing surface accessibility and hydration dynamics. Spherical nanoparticles with smooth and compact surfaces generally undergo more uniform hydration and degradation, supporting reproducible release profiles. In contrast, anisotropic or non-spherical geometries (e.g., rods or disks) may alter surface area distribution and local thickness, affecting water penetration and internal transport pathways [56,57]. Calis et al. [56] synthesized spherical, rod-shaped, and elliptical disk-shaped PLGA nanoparticles and evaluated their in vitro and in vivo behavior. Despite comparable size distributions and drug loading, spherical particles exhibited significantly higher cumulative release of encapsulated human serum albumin compared to rod and disk geometries. These findings suggest that particle shape alters surface accessibility and diffusion pathways, thereby modulating release kinetics independently of polymer composition. Wu et al. [58] demonstrated emulsion electrospray as an efficient method for nanoencapsulating water-soluble drugs, (bovine serum albumin (BSA)) into PLGA microspheres, achieving high EE (>80%) with tunable morphology and release profiles (Figure 3). By varying the aqueous-to-organic phase volume ratio (V_w_/V_o_), they controlled drug nanodroplet distribution—from discrete droplets at low ratios to interconnected phases at high ratios—leading to biphasic release kinetics: initial burst from surface-accessible drug followed by diffusion-controlled sustained release.

Surface porosity plays a particularly critical role. Increased porosity shortens transport path lengths and enhances matrix permeability, accelerating early-stage release. Highly porous or sponge-like structures often display elevated burst release due to rapid drug transport through interconnected aqueous channels, whereas dense, nonporous matrices restrict transport and prolong release [56].

#### 2.3.3. Surface Charge

The surface charge of particles plays a critical role in their interaction with cells and subsequent uptake. Nano- and microparticles made from uncoated PLGA typically exhibit negative surface charge at physiological pH, due to carboxyl groups that arrange at the liquid/particle [59]. The particle surface can be modified by coating with cationic polyelectrolytes through ionic interactions. However, the adsorption of polyelectrolytes onto the surface of particles in suspension is complex and influenced by various parameters. The structure and packing of the adsorbed layer is widely determined by the particle surface charge density, the charge density of polyelectrolyte, as well as by the pH and ionic strength of surrounding medium [60].

The coating of negatively charged particles has been found to be advantageous for several applications. The positive charges imparted at the particle surface, for example, efficiently complex anionic macromolecules such as plasmid DNA, and this has received considerable interest for the formulation of vaccines. Moreover, surface decoration with polycations is considered a straightforward strategy to enhance the adhesion of particles to cell membranes [61]. For example, Luidweg et al. [62] synthesized ciprofloxacin-loaded PLGA nanoparticles combined with positively charged polymers, Eudragit^®^ RS100 and RL100, to modify the surface charge of nanoparticles. This surface modification generated positively charged particles capable of interacting with the anionic mucins present in the mucus layer of the tear film, thereby improving mucoadhesion and potentially enhancing ocular drug delivery.

To make use of these potential benefits, natural, processed natural, and synthetic polyelectrolytes have been employed for the coating of negatively charged PLGA nano- and microparticles. These include chitosan [63,64,65,66,67,68,69], gelatine [70,71,72], diethylaminoethyl dextran, poly(ethylene imine) (PEI) [73], and polyinosinic:polycytidylic acid (poly(I:C)) [74,75]. Some studies have addressed the usability of chitosan for the surface modification of nano- and microparticles made from PLGA. For example, chitosan-coated PLGA nanoparticles have also offered an alternative method for delivery of bevacizumab to the posterior segment of the eye. Due to its positive surface charge, chitosan-coated PLGA particles offer enhanced adhesion to the negatively charged cell membrane and confer longer drug release [76].

An alternative and quite promising approach has relied on the coating of pre-formed PLGA particles with multifunctional PLL-g-PEG polymers [77].

Various properties of nanomaterials such as size, composition, surface charge, hydrophobicity or hydrophilicity affect their interactions with biological components. Adsorption of proteins on the nanoparticle surface has a major impact on pharmacokinetics and pharmacodynamics influence the fate of nanoparticles in body. One of the most essential parameters in adsorption is the surface charge of the nanoparticles. Menon et al. [78] investigated the interaction of PLGA nanoparticles with plasma proteins, cell and haematological components. PLGA nanoparticles with similar particle size but different surface charge were prepared by employing PEI, PVA and casein surface passivation. Their results showed that the amount of plasma protein binding varied with surface charge of the particles and positively charged particles exhibiting the highest protein adsorption and consequently higher cellular accumulation.

Although significant progress has been achieved in tuning particle size, shape, and morphology through microfluidic and electrohydrodynamic approaches, translating these physical parameters into predictable biological outcomes remains a major challenge. While controlling processing parameters allows precise tailoring of micro- and nanoparticle morphology, a deeper understanding of how such parameters collectively influence degradation, cellular uptake, and pharmacokinetics is still not fully understand.

## 3. Electrospray Technique for PLGA Nanoparticle Synthesis

### 3.1. Electrospray Mechanism

**Figure 4:**
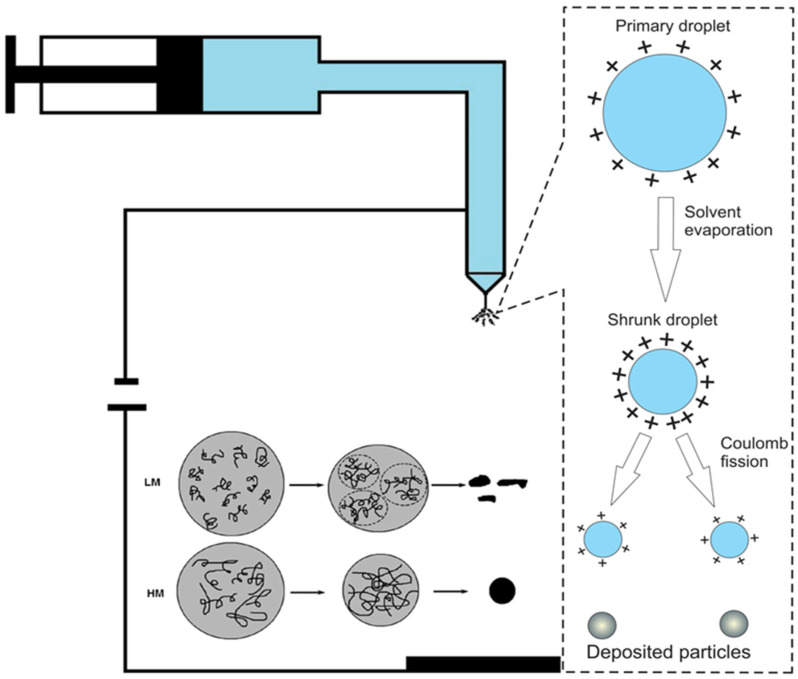
Schematic representation of the electrospray process. A polymer solution is delivered through a needle under a high-voltage electric field, forming a charged primary droplet. As the droplet travels toward the collector, solvent evaporation leads to droplet shrinkage and charge accumulation, followed by Coulomb fission into smaller droplets. This process ultimately results in the formation and deposition of solid nanoparticles on the collector. Variations in polymer concentration influence chain entanglement and final particle morphology. Blue regions represent the polymer solution/droplets, “+” symbols indicate surface charges, and arrows illustrate the droplet evolution and particle formation process. LM and HM denote low and high-molecular-weight conditions, respectively. Adapted with permission from [13] © 2016 American Pharmacists Association^®^. Published by Elsevier Inc. (Amsterdam, The Netherlands). All rights reserved.

Electrospray, also referred to as electrohydrodynamic atomization, is a widely used technique for the fabrication of polymeric micro- and nanoparticles in pharmaceutical and biomedical applications. The process involves the dispersion of a polymer solution into fine charged droplets under the influence of a strong electric field. A polymer solution is delivered at a controlled flow rate through a metallic capillary using a syringe pump, while a high voltage is applied between the needle and the grounded collector, generating the electrostatic forces required for droplet formation. As the droplets travel toward the collector, solvent evaporation occurs, and solid polymeric particles are deposited on the substrate [17,79]. When the electric potential is applied, electrical charges accumulate at the surface of the liquid meniscus emerging from the needle tip. The electrostatic pressure generated by these charges counteracts the surface tension of the liquid, deforming the droplet into a conical structure known as the Taylor cone. Once the electrostatic stress exceeds the restoring force of surface tension, a fine charged jet is emitted from the apex of the cone. This phenomenon, known as charge-driven atomization, marks the transition to the cone–jet regime and represents the fundamental mechanism governing electrospray particle formation [11]. The emitted jet subsequently undergoes elongation and fragmentation due to Coulombic repulsion between surface charges. Through capillary breakup and solvent evaporation, the jet disintegrates into uniformly sized droplets that ultimately solidify into micro- or nanoparticles. The stability of the cone–jet mode depends on a balance between electric field strength, flow rate, solution conductivity, viscosity, and polymer concentration. Under optimal conditions, a stable jet is produced that generates monodisperse particles with controllable size and morphology [80]. The electrospray apparatus typically consists of four main components: a high-voltage power supply, a syringe pump to control the solution flow rate, a metallic needle connected to the voltage source, and a grounded collector where particles are deposited (Figure 4) [13]. Variations in collector configuration (e.g., flat plates or rotating collectors) may influence particle deposition patterns and morphology.

### 3.2. Key Operational Parameters

#### 3.2.1. Solution Parameters

The physicochemical properties of the polymer solution strongly influence droplet formation and particle characteristics during electrospraying. Important solution parameters include polymer concentration, viscosity, surface tension, electrical conductivity [81], solvent type [82], and polymer molecular weight [13]. These parameters collectively determine jet stability and droplet breakup behaviour. Polymer concentration plays a particularly important role because it directly affects solution viscosity and chain entanglement. Increasing polymer concentration generally increases viscosity and surface tension, which may lead to the formation of larger particles. However, excessively high concentrations can promote aggregation or even transition the process from electrospraying to electrospinning, producing fibers rather than discrete particles. Conversely, very low concentrations may result in unstable jets and irregular droplet formation due to insufficient polymer chain entanglement [81]. The choice of solvent also significantly affects the electrospray process. Solvent properties such as boiling point, vapor pressure, conductivity, and polymer solubility determine droplet evaporation rates and particle solidification kinetics. Solvents with excessively low boiling points may evaporate too rapidly and cause needle clogging, while solvents with high boiling points may slow drying and lead to larger particles due to incomplete solvent removal during flight [83].

#### 3.2.2. Process Parameters

The process parameters of the electrospray setup further determine the final particle characteristics. These include applied voltage, solution flow rate, and the distance between the needle and collector [84]. The applied voltage represents the primary driving force of the electrospray process, as it determines the strength of the electric field responsible for jet formation. Increasing the voltage generally enhances electrostatic forces and promotes the generation of smaller droplets. However, excessively high voltages may destabilize the cone–jet mode, leading to multiple jets, particle aggregation, or broad size distributions [13]. The flow rate controls the volume of liquid delivered to the needle tip per unit time and therefore influences droplet size and solvent evaporation. Higher flow rates typically produce larger particles due to insufficient drying time, whereas lower flow rates favor the formation of smaller and more uniform nanoparticles [81]. The needle-to-collector distance, often referred to as the flight distance, determines the residence time available for solvent evaporation during droplet travel. Short distances may result in incomplete solvent evaporation and particle deformation, while excessively long distances can produce porous structures due to rapid solvent evaporation. Maintaining an optimal spacing is therefore essential for achieving uniform particle morphology [81].

#### 3.2.3. Environmental Parameters

In addition to solution and operational parameters, environmental conditions can significantly influence electrospray outcomes. Temperature, humidity, airflow, and chamber pressure affect solvent evaporation rates and therefore impact particle morphology and size distribution [85]. Elevated temperatures combined with low humidity typically accelerate solvent evaporation and promote the formation of smaller and more uniform particles. Conversely, high humidity levels slow the drying process and may lead to larger or less uniform particles. Airflow within the electrospray chamber can also influence droplet transport and drying dynamics, affecting particle deposition and morphology [86].

### 3.3. Comparison with Other Nanoparticle Fabrication Methods

A variety of fabrication strategies have been developed for polymeric nanoparticles in drug delivery, including emulsion–solvent evaporation, nanoprecipitation, solvent diffusion, spray drying, and layer-by-layer assembly. While these methods are widely used for biodegradable polymers such as PLA, PLGA, and PCL, they often involve multiple processing steps, surfactants, and significant amounts of organic solvents, which can affect reproducibility, particle size control, and residual solvent content [6]. Process yield is also a critical consideration for practical applications. Conventional emulsion-based methods typically exhibit lower yields due to material loss during processing, whereas techniques such as spray drying and electrospray can achieve higher efficiencies under optimized conditions [87]. Electrospray offers a distinct approach based on electrohydrodynamic atomization, enabling the production of particles with narrow size distributions and tunable morphology under relatively mild conditions and with minimal surfactant use. However, each technique presents specific advantages and limitations depending on formulation requirements and scalability considerations. A comparative overview of these methods is provided in Table 1 [88].

## 4. Dual Drug Loading and Release Mechanisms via Electrosprayed PLGA Nanoparticles

The co-delivery of multiple therapeutic agents using PLGA nanoparticles has emerged as a promising strategy to enhance therapeutic efficacy, especially in cancer treatment and immunotherapy [89]. Combining drugs with complementary mechanisms of action can produce synergistic therapeutic effects, reduce drug resistance, and enable simultaneous targeting of multiple biological pathways [90]. Electrospray-based fabrication offers a particularly attractive platform for such systems because electrohydrodynamic atomization enables precise control over particle architecture and drug distribution, facilitating the design of carriers with tailored and potentially sequential release behavior. These characteristics facilitate the design of nanocarriers capable of loading multiple drugs and controlling their release kinetics. Table 2 summarizes electrosprayed PLGA nanoparticles encapsulating single or dual therapeutic agents developed for the treatment of various diseases.

### 4.1. Encapsulation Architectures for Dual Drug Delivery

**Figure 5:**
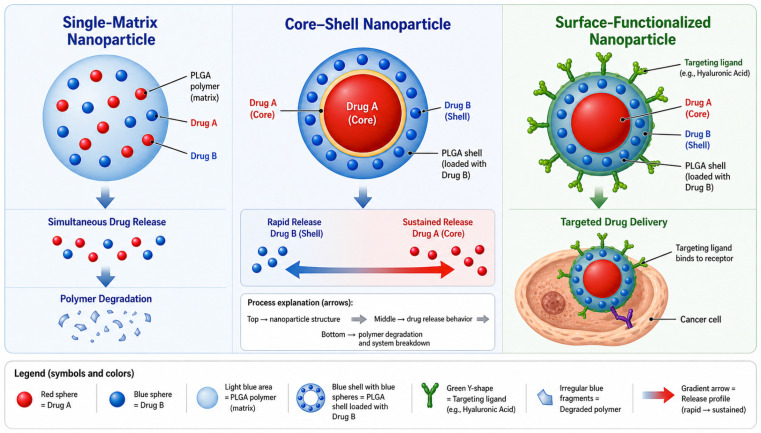
Different encapsulation strategies for dual-drug delivery (created by the authors).

Several structural strategies have been developed to enable the co-encapsulation of multiple drugs within electrosprayed PLGA nanoparticles. Single-matrix nanoparticles represent the simplest design, in which both therapeutic agents are dispersed within the same polymer matrix. In this configuration, drugs are released simultaneously as the polymer undergoes hydration, diffusion, and degradation. This approach is particularly suitable when the therapeutic agents have compatible physicochemical properties and similar release requirements. However, controlling individual release kinetics can be challenging because both drugs are exposed to the same polymer environment [116]. A more sophisticated design involves core–shell nanoparticles, which are typically produced using coaxial electrospray configurations [117]. In these systems, one drug is localized in the particle core while the second drug is incorporated within the outer polymer shell. This architecture enables spatial separation of therapeutic agents and allows the design of sequential or staged drug release profiles. Core–shell PLGA nanoparticles have been widely explored for combination cancer therapy, where one agent may be released rapidly to initiate therapeutic activity, while the second drug is released gradually to sustain treatment [107]. Another emerging strategy involves surface-functionalized nanocarriers, where the nanoparticle surface is modified with targeting ligands or functional polymers to enhance tumor accumulation and cellular uptake. For example, the incorporation of targeting moieties such as hyaluronic acid can promote receptor-mediated uptake in cancer cells that overexpress CD44 receptors, improving therapeutic selectivity and intracellular drug delivery [118]. Figure 5 shows different encapsulation strategies for dual-drug delivery.

### 4.2. Challenges in Co-Delivery of Bioactive Agents

Despite the significant therapeutic potential of dual-drug PLGA nanoparticles, several formulation challenges must be addressed to achieve the effective co-delivery of bioactive agents such as chemotherapeutic or immunotherapeutic agents. One major difficulty arises from the distinct physicochemical properties of the encapsulated drugs. Many chemotherapeutic agents are small, hydrophobic molecules, whereas immunotherapeutic agents such as peptides, proteins, or antibodies are typically hydrophilic and structurally fragile. These biomolecules are particularly sensitive to organic solvents, shear stress, and interfacial denaturation during nanoparticle fabrication. For instance, PLGA nanoparticles encapsulating CMV antigen peptides (pp65 and IE-1) require mild processing conditions to preserve antigen integrity and immunogenic activity [102]. Another important challenge involves the precise control of simultaneous versus sequential drug release. In combination therapy, optimal therapeutic outcomes often depend on delivering drugs in a specific temporal sequence. Core–shell nanoparticle architectures fabricated by coaxial electrospray provide a promising strategy for achieving staggered release profiles, where the shell-loaded drug is released rapidly while the core-encapsulated drug is released in a sustained manner. However, achieving reproducible release kinetics requires careful optimization of several parameters, including polymer composition, solvent systems, layer thickness, and electrospray operating conditions [15]. For example, core–shell nanoparticles containing PTX and imatinib have demonstrated sequential release behavior through controlled spatial distribution of the drugs within the particle structure [107]. A further challenge is the prevention of premature drug leakage and excessive burst release. The rapid release of surface-associated drug molecules can reduce the duration of therapeutic activity and compromise controlled-release performance. EE and particle stability are strongly influenced by electrospray parameters such as solvent choice, polymer concentration, and solution flow rate [14]. Optimized formulations have demonstrated high EE, such as PTX–imatinib systems with efficiencies of 94.4% and 97.5% [107], as well as DOX-loaded nanoparticles with efficiencies exceeding 85% [104], highlighting the potential of electrospray-based fabrication to produce stable dual-drug delivery systems when process parameters are carefully controlled.

### 4.3. Role of Stimuli-Responsive PLGA Formulations

Stimuli-responsive PLGA nanoparticles have emerged as an effective strategy for improving the selectivity and therapeutic efficiency of anticancer drug delivery [119]. These systems exploit specific characteristics of the tumor microenvironment, such as acidic pH, enzymatic activity, or intracellular redox gradients, to trigger controlled drug release at the target site [120]. For example, pH-responsive formulations can accelerate drug release in the slightly acidic conditions of tumor tissues or intracellular compartments [119], while enzyme- or redox-responsive linkers enable localized release following cellular uptake [121]. Such responsive systems are particularly advantageous for dual-drug delivery, where the therapeutic outcome may depend on controlled or sequential release of multiple agents. For instance, an initial release of a chemotherapeutic drug may induce tumor cell damage and antigen release, followed by delayed release of immunomodulatory molecules that enhance anti-tumor immune responses [122]. Although most current electrosprayed PLGA nanoparticles focus on delivering either chemotherapeutic drugs or immunotherapeutic peptides individually, integrating stimuli-responsive mechanisms into dual-drug electrosprayed systems represents a promising strategy for improving combination chemo-immunotherapy [123]. Stimuli-responsive PLGA-based nanocarriers have been developed by incorporating responsive polymers or cleavable linkers into the nanoparticle structure. For example, dual pH/redox-responsive nanoparticles containing disulfide-linked poly(β-amino ester) shells and PLGA cores have demonstrated triggered intracellular drug release in response to acidic and reductive tumor microenvironments [123].

## 5. Mathematical Modeling for PLGA Nanoparticles in Electrospray

Mathematical modeling plays a crucial role in understanding and optimizing the electrospray fabrication of PLGA nanoparticles. Modeling approaches can be broadly categorized into three types: (i) theoretical descriptions of electrospray jet formation [124], (ii) models drug release from PLGA particles [125], and (iii) advanced numerical simulations and data-driven optimization techniques such as finite element modeling (FEM) and machine learning (ML) [126].

### 5.1. Modeling of Nanoparticle Formation

#### 5.1.1. Electrohydrodynamic Theory

The electrospray process is governed by electrohydrodynamic forces acting on a conductive liquid subjected to a strong electric field. When a high voltage is applied between the capillary and the collector, electrostatic stresses deform the liquid meniscus into a conical structure known as the Taylor cone, characterized by a full cone angle of approximately 98°. Once the electrostatic force exceeds the restoring force of surface tension, a fine liquid jet is emitted from the cone apex, producing the stable cone–jet regime responsible for generating charged droplets. Scaling relationships derived from electrohydrodynamic theory are commonly used to estimate droplet size and charge. In simplified form, the droplet diameter can be expressed as in Equation (1):

(1) $$d∼(\frac{\gamma Q}{\varepsilon_{0}V}{)}^{1/3}$$

where Q is the liquid flow rate, *V* is the applied voltage, *γ* is the surface tension, and *ε*_0_ is the permittivity of free space. More advanced theoretical descriptions incorporate electrokinetic effects through models such as the leaky-dielectric model and the Poisson–Nernst–Planck equations, which describe charge transport, ionic migration, and electric field distribution on the droplet surface [127].

#### 5.1.2. CFD and Multiphysics Simulations

Multiphase Computational Fluid Dynamics (CFD) simulations are widely used to model the electrospray process by coupling fluid dynamics, electrostatics, heat transfer, and solvent evaporation. These models typically solve the Navier–Stokes equations together with electrostatic field equations and mass transport models to describe the evolution of the liquid jet and droplet formation. Interface-tracking methods such as the Volume of Fluid (VOF) or level-set approaches are often employed to capture the deformation and breakup of charged droplets. Through these multiphysics simulations, the transition from a charged liquid droplet to a solidified polymer particle can be predicted [128]. Such models are particularly useful for estimating the final particle morphology (solid, porous, or core–shell structures), especially in coaxial electrospray systems, where different evaporation rates of the core and shell solutions strongly influence nanoparticle architecture [129,130].

#### 5.1.3. Statistical Process Optimization

Statistical design methods, such as the Taguchi approach, are frequently employed for the multivariable optimization of electrospray process parameters, including applied voltage, solution flow rate, polymer concentration, needle-to-collector distance, and solvent composition [128]. By systematically evaluating the influence of multiple factors with a limited number of experiments, Taguchi designs enable efficient identification of optimal operating conditions. When combined with numerical simulations or response surface modeling, these statistical approaches help define the operational window for a stable cone–jet regime and facilitate the production of nanoparticles with controlled size distribution and morphology [131,132].

### 5.2. Mathematical Models for Drug Release Kinetics

#### 5.2.1. Diffusion-Based Models

During the initial stage of drug release, transport is primarily governed by diffusion through the polymer matrix or aqueous pores. This process is commonly described by Fick’s second law, which accounts for radial diffusion in spherical or core–shell geometries:

(2) $$\frac{\partial c}{\partial t}=D\left(\frac{1}{r^{2}}\frac{\partial }{\partial r}\left(r^{2}\frac{\partial c}{\partial r}\right)\right)$$

where c represents drug concentration, *t* is time, *r* is the radial position, and *D* is the diffusion coefficient.

Simplified analytical models are often used to interpret experimental data. The Higuchi model predicts that the cumulative drug release is proportional to the square root of time (∼t^1/2^), reflecting diffusion-controlled transport. In addition, the Korsmeyer–Peppas model is widely applied to fit release data and distinguish between Fickian diffusion and anomalous transport mechanisms [133].

#### 5.2.2. Degradation-Controlled Models

At later stages, drug release is increasingly influenced by polymer degradation. PLGA undergoes bulk erosion, where hydrolytic cleavage of ester bonds leads to a progressive decrease in molecular weight and the formation of pores within the matrix. This process enhances drug mobility and alters diffusion pathways. Empirical models such as the Hixson–Crowell equation describe changes in particle size and surface area during degradation, while more advanced mechanistic models incorporate autocatalytic effects arising from the accumulation of acidic degradation products. These models are particularly useful for predicting long-term release behavior and understanding heterogeneous degradation within the particle core [134].

#### 5.2.3. Hybrid and Core–Shell Models

In many PLGA systems, drug release is governed by a combination of diffusion and degradation mechanisms. Hybrid models integrate these processes to more accurately describe release kinetics over time. This approach is particularly relevant for core–shell nanoparticles, where spatial separation of drugs enables distinct release profiles. In such systems, the shell-associated drug is typically released rapidly via diffusion, while the core-encapsulated drug exhibits sustained release governed by polymer degradation. Advanced models further incorporate time-dependent changes in porosity, swelling, and internal structure, enabling prediction of sequential drug release behavior and improved correlation with experimental in vitro data [135].

### 5.3. Finite Element Modeling (FEM) and Artificial Intelligence-Based Optimization

#### 5.3.1. FEM for Coupled Mass, Heat, and Electric Fields

FEM enables the numerical solution of coupled transport equations governing mass transfer, heat transfer, and electrostatic fields during electrospray and subsequent particle evolution. These models are capable of simulating solvent evaporation, charge distribution, and polymer solidification within charged droplets, thereby predicting internal drug distribution and final particle morphology [136]. FEM is particularly valuable for the rational design of PLGA-based delivery systems, as it allows prediction of long-term release behavior based on initial particle structure and composition [137].

#### 5.3.2. ML Approaches for Process Optimization

ML approaches, including artificial neural networks, random forests, and decision tree algorithms, have recently emerged as powerful tools for predicting electrospray outcomes [126,138]. These models can correlate process parameters such as voltage, flow rate, and polymer concentration with particle size, morphology, and size distribution. By integrating experimental datasets with data-driven modeling, ML enables rapid optimization of process conditions while significantly reducing trial-and-error experimentation. Recent studies have also applied ML to model plume dynamics and droplet evolution, demonstrating the potential of data-driven approaches for real-time process control and optimization. For example, J.D. Breddan et al. [139] applied ML approach to model electrospray plume dynamics, using simulated particle data to predict how droplets behave from emission to final particle properties. The authors generated computational electrospray datasets and trained multiple ML models (e.g., Random Forest, Support Vector Regression, neural networks) to establish relationships between initial emission parameters (such as velocity, charge, and direction) and final particle characteristics. Their results show that ML can successfully predict key outcomes like plume angle and particle behavior, while also identifying which physical parameters have the strongest influence (e.g., particle charge and emission velocity).

#### 5.3.3. Simulation in Biological Systems

Beyond particle formation, FEM and CFD simulations are increasingly applied to predict nanoparticle transport, deposition, and distribution within biological environments, including pulmonary, tumor, and tissue systems [140]. These models incorporate fluid dynamics, diffusion, and biological barriers to estimate particle fate following administration. Such simulations are particularly relevant for inhalation therapies and implantable delivery systems, where they provide a quantitative framework for dose localization, tissue penetration, and therapeutic efficacy [141].

### 5.4. Limitations and Future Directions

Despite substantial advances in mathematical modeling of electrosprayed PLGA nanoparticles, several critical limitations remain, particularly regarding the predictive capability of current models for in vivo behavior. Although CFD and FEM models have demonstrated strong performance in describing electrospray physics, droplet formation, and in vitro drug release, they often fail to accurately predict in vivo pharmacokinetics. This limitation primarily arises from the simplified assumptions used in these models, which neglect key biological factors such as protein adsorption (protein corona formation), immune system interactions, tissue heterogeneity, and physiological transport barriers [142]. Moreover, most current models do not incorporate biological clearance mechanisms, such as renal filtration, hepatic metabolism, and uptake by the mononuclear phagocyte system (MPS), all of which play a critical role in determining nanoparticle biodistribution and fate after administration. Consequently, a significant discrepancy often exists between model predictions and experimental in vivo outcomes.

Additional limitations include the incomplete representation of key physicochemical processes, such as polymer swelling, pore formation, cracking, and autocatalytic degradation, which strongly influence drug release behavior [123,143]. Furthermore, the development of robust ML models is constrained by the limited availability of large, high-quality experimental datasets. More comprehensive datasets covering diverse PLGA formulations and processing conditions are required to improve predictive accuracy [144]. Modeling of multi-layered and core–shell systems also remains challenging, as interfacial interactions and their effects on drug release are not yet fully understood or quantitatively described [111].

To address these challenges, future modeling efforts should focus on integrating biological parameters into existing physical frameworks. In particular, coupling transport and degradation models with biological clearance rates and biodistribution data could significantly improve predictive capability. The development of hybrid modeling approaches that combine physics-based methods (e.g., FEM) with data-driven techniques (e.g., ML trained on in vivo datasets) represents a promising direction. Furthermore, the incorporation of physiologically based pharmacokinetic (PBPK) models into CFD/FEM frameworks could enable more realistic simulation of nanoparticle transport, systemic circulation, tumor accumulation, and organ-specific clearance. These integrative approaches could improve the connection between in vitro design and in vivo performance [145]. Achieving this goal will require close collaboration between experimental and computational researchers to generate high-quality in vivo datasets for model calibration and validation [146].

## 6. Applications of Electrospray-Based PLGA NPs in Cancer Therapy & Immunotherapy

PLGA nanoparticles are widely explored in oncological drug delivery due to their ability to modulate drug distribution and release profiles in complex biological environments. By enabling controlled and sustained delivery of chemotherapeutic agents, these systems can enhance intratumoral drug retention while limiting off-target exposure. This is particularly relevant for drugs with narrow therapeutic windows, where precise control over release kinetics can influence treatment efficacy and safety. In addition, the versatility of PLGA-based platforms allows for the incorporation of targeting ligands, imaging agents, or combination therapies, supporting the development of multifunctional nanomedicines for cancer treatment [9]. Recent advances in fabrication technologies, particularly electrospray, have greatly improved the potential of PLGA systems. This method enables precise control over particle size, morphology, EE, and multi-drug loading. Electrospray-fabricated PLGA nanoparticles can achieve high drug loading and stable incorporation of hydrophobic molecules, resulting in controlled release profiles without burst effects. Additionally, this technique supports scalable production while minimizing solvent residues [7]. Such structural tunability is especially important in cancer therapy, as treatment effectiveness often depends on optimizing drug localization, release kinetics, and interactions with the tumor microenvironment. From a biomedical perspective, PLGA nanoparticles are versatile in both chemotherapy and immunotherapy. As drug carriers, they increase the concentration of anticancer agents at tumor sites, extend systemic circulation time, and improve therapeutic outcomes compared to free drugs [147]. PLGA nanoplatforms not only deliver cytotoxic drugs but also support immunomodulation by carrying antigens, adjuvants, or immune-targeting molecules that enhance immune responses or modify signaling pathways in the tumor microenvironment [9]. This dual functionality allows for integrated chemo-immunotherapeutic strategies that combine direct tumor cytotoxicity with immune activation, resulting in synergistic anticancer effects. Furthermore, nanoparticle surface engineering and compositional tunability enhance targeted delivery methods, such as passive accumulation in tumors and active receptor-mediated targeting. These advancements increase the clinical relevance of PLGA nanoparticles by improving therapeutic specificity, reducing off-target toxicity, and enabling combination treatment strategies [148]. This section explores key applications of electrospray-based PLGA nanoparticles in cancer therapy and immunotherapy. It highlights case studies involving chemotherapeutic agents and immune-modulating substances, followed by a discussion of targeting strategies that enhance their potential for precision oncology.

### 6.1. Case Studies & Clinical Relevance

#### 6.1.1. PLGA Nanoparticles for Chemotherapy

PLGA nanoparticles have been extensively studied as carriers for chemotherapeutic drugs like PTX and DOX, offering significant advantages over traditional free-drug administration. Encapsulating PTX in PLGA nanoparticles enhances the drug’s solubility and protects it from rapid degradation in biological environments. PLGA nanosystems enhance therapeutic efficacy by enabling sustained release and preferential accumulation in tumor tissue through the enhanced permeability and retention (EPR) effect. This approach reduces systemic toxicity and improves the therapeutic index compared to free drugs, as demonstrated in preclinical and clinical studies [149,150]. For example, PLGA nanosystems have shown improved bioavailability, reduced cardiotoxicity with DOX, and decreased solvent-related hypersensitivity with PTX [151,152]. Compared to traditional PLGA nanoparticle fabrication methods like emulsion–solvent evaporation and nanoprecipitation, electrospray techniques provide superior control over particle size distribution, nanoparticle architecture (including core–shell designs), and surface properties, while avoiding high-energy mixing and minimizing the need for surfactant stabilizers. Electrospray has been shown to produce monodisperse particles with improved EE and customizable release profiles, achieved through precise droplet formation and solvent evaporation dynamics. In contrast, conventional methods typically result in wider size distributions and lower EE due to their dependence on mechanical emulsification and surfactants [118,153].

Recent electrospray-based PLGA formulations demonstrate tunable particle architecture and drug loading capacity. A core–shell PLGA nanoparticle system created through coaxial electrospraying effectively encapsulated DOX with over 80% efficiency and demonstrated reduced burst release. The sustained release profile (~69% over 144 h) significantly increased cytotoxicity against MCF-7 breast cancer cells compared to matrix PLGA nanoparticles and free DOX, resulting in a markedly lower IC_50_. The polymer shell protected drug molecules from premature degradation and enabled diffusion-controlled release, underscoring the potential of electrospray-engineered nanostructures for precision chemotherapy delivery [104].

Electrospray-derived PLGA submicron particles produced via dual-capillary electrohydrodynamic atomization show that both particle size and internal structure significantly affect drug release kinetics. In these particles, drug release is mainly governed by solvent diffusion and water permeation through the PLGA matrix. Notably, increasing particle size leads to a marked reduction in release rate. These findings provide mechanistic insights indicating that electrospray processing enables precise tuning of chemotherapeutic release profiles by manipulating particle morphology. This capability is essential for optimizing therapeutic windows and reducing systemic toxicity [105].

Electrospray-produced particles ranging from nanometers to micrometers show size-dependent diffusion-controlled release and structural stability, making them suitable for encapsulating hydrophobic agents like PTX. These findings establish a technological platform for designing PTX-PLGA nanoparticle systems, emphasizing the method’s adaptability for poorly soluble chemotherapeutics. Literature on PLGA chemotherapy carriers shows that PTX encapsulation significantly improves pharmacokinetics and antitumor efficacy. For example, modified PLGA nanoparticles, such as cyclodextrin-functionalized PLGA, have shown increased blood residence time for PTX, improved tumor accumulation, and enhanced therapeutic effectiveness in vivo compared to conventional formulations like Taxol^®^ [152].

Table 3 provides an overview of representative PLGA nanoparticle systems used in chemotherapy (DOX and PTX), focusing on electrospray and advanced fabrication techniques. The key parameters summarized include fabrication method, particle size, drug loading, release profile, experimental model, and therapeutic outcomes. The studies indicate that electrospray fabrication and functional modification strategies enhance nanoparticle stability, bioavailability, tumor targeting, and therapeutic efficacy compared to free drugs. Clinically, these advancements suggest that electrospray-engineered PLGA nanoparticles can optimize chemotherapy regimens by minimizing systemic side effects and improving treatment outcomes in solid tumors.

#### 6.1.2. PLGA Nanoparticles for Immunotherapy

PLGA nanoparticles have been extensively studied as carriers for cancer immunotherapy due to their biodegradability, biocompatibility, and adjustable release properties. These features are crucial for effectively delivering immunotherapeutic agents such as cytokines, nucleic acids, and immune checkpoint modulators. Recent reviews suggest that PLGA platforms significantly enhance antigen presentation, reprogram tumor-associated macrophages, and facilitate synergistic effects in checkpoint blockade by improving payload stability, local concentration, and sustained exposure in vivo. These mechanisms ultimately lead to a pronounced amplification of antitumor immune responses when compared to free immunotherapeutics [4,5,6]. PLGA nanoparticles allow controlled spatiotemporal delivery of immunomodulators, minimizing systemic toxicity and prolonging circulation time. These features are especially important for delicate cytokines or large biomolecules that are rapidly cleared when administered in their free form in vivo [9].

Immunotherapeutic applications of PLGA nanoparticles have been demonstrated in various studies. For example, PLGA nanoparticles co-delivering PD-L1 and PD-1 small interfering RNA (siRNA) significantly enhanced host immune responses by restoring CD8^+^ T cell functionality and promoting cytotoxic activity in tumor models, outperforming antibody-based checkpoint blockade in preclinical settings [165]. Similarly, in pancreatic cancer models, siRNA targeting PD-L1 encapsulated in PLGA nanoparticles significantly increased CD8^+^ T-cell infiltration and inhibited tumor progression. This finding suggests that PLGA-mediated delivery of immune checkpoint silencing agents can overcome the limitations of antibody delivery and enhance immune activation [166]. In addition to checkpoint targeting, PLGA micro/nanoparticles designed for delivering anti-PD1 antibodies and antigens have induced significant resident memory CD8^+^ T cell responses, facilitating tumor rejection and reversing T cell exhaustion in hepatocellular carcinoma models [167]. These findings demonstrate that PLGA carrier systems can enhance antigen uptake, promote dendritic cell maturation, and boost effector T cell activation, which are crucial for the efficacy of cancer immunotherapy.

Cytokine delivery using PLGA carriers is a promising strategy for immune modulation. Encapsulating cytokines within biodegradable polymer matrices addresses challenges related to rapid degradation and excessive systemic exposure, while allowing for sustained release, which may enhance both innate and adaptive immune responses. Experimental studies show that polymeric cytokine delivery systems can increase interferon-γ production, improve antigen presentation, and enhance T-cell-mediated cytotoxicity compared to soluble cytokine administration. These findings highlight the potential of PLGA nanoparticle-mediated cytokine delivery to strengthen antitumor immunity [168,169]. For example, IL-12-encapsulated PLGA nanoparticles targeting CD8^+^ T cells showed sustained cytokine delivery and promoted the expansion, activation, and cytotoxic activity of T lymphocytes compared to free cytokine administration [170]. Moreover, biomimetic and lipid/PLGA hybrid nanocomplexes that incorporate immune checkpoint inhibitors (e.g., PD-L1 peptide inhibitors) with additional therapies (such as indoleamine 2,3-dioxygenase inhibition and photodynamic therapy) have shown that targeted PD-L1 blockade within a PLGA platform can effectively recruit cytotoxic T cells and induce strong immunogenic cell death in CT26 tumor-bearing mice, highlighting the potential of combinatorial immunotherapy [171].

Despite the promising outcomes of immunotherapy using PLGA systems, the literature on electrospray-specific case studies for delivering immune payloads is still relatively limited. In contrast to chemotherapy agents like DOX and PTX, which have been widely used in electrospray techniques to create monodisperse, core–shell, and tunable delivery systems, immunotherapeutic payloads often consist of sensitive macromolecules (e.g., cytokines or antibodies) that present challenges for electrohydrodynamic processing [10,102]. Encapsulating proteins and peptides in hydrophobic polymer matrices like PLGA presents challenges due to the hydrophilic nature of many biologics. These molecules are prone to denaturation, aggregation, or loss of bioactivity when exposed to organic solvents, strong electric fields, or interfacial stresses during particle formation [172]. In electrospray, the rapid formation of charged droplets under strong electric fields and the use of volatile organic solvents to dissolve PLGA can stress protein conformations, complicating the preservation of their native structure and bioactivity. Consequently, these factors result in low drug loading, poor EE, and reduced protein activity, ultimately limiting the clinical translation of protein-loaded polymer nanoparticles [172,173]. While some electrospray studies have shown that this technique can effectively encapsulate peptides and stimulate the immune system (e.g., peptide-loaded PLGA nanoparticles enhancing antigen-specific CD8^+^ T-cell proliferation), this work focused on relatively small peptides rather than full-length cytokines or antibodies [102]. Research on electrospray protein encapsulation in biodegradable polymer systems shows that bioactivity can be preserved (e.g., sustained release of BSA with maintained structural integrity) when conditions are appropriately optimized. However, these require strict control of process parameters and often need precisely calibrated additives [174]. In contrast, conventional techniques such as double emulsion and nanoprecipitation are more widely used for formulating immunotherapeutic payloads. These methods enable gentler aqueous processing and offer greater flexibility for incorporating protein stabilizers and excipients that protect biological structures. Consequently, they are more frequently employed in procedures related to immune modulation and vaccine development [173].

### 6.2. Targeted Drug Delivery Strategies

Targeted drug delivery has become a key design principle in developing nanocarriers for cancer therapy and immunotherapy. This approach aims to enhance the therapeutic index and reduce off-target toxicity by improving drug accumulation at pathological sites. In the context of electrospray-fabricated PLGA nanoparticles, targeting strategies are crucial due to the ability of electrospray processing to provide precise control over particle size distribution, morphology, and encapsulation architecture. These factors directly affect biodistribution and cellular interactions. The structural tunability of this technique has led to the development of polymeric carriers that can deliver chemotherapeutic agents, immune modulators, and combination payloads, enhancing pharmacokinetic behavior and tumor localisation [104]. Comprehensive reviews show that PLGA-based nanocarriers enable controlled release, exhibit biodegradability, and have significant clinical potential in oncology. This is due to their ability to encapsulate various therapeutic agents while degrading into biocompatible metabolites [16,118].

In cancer therapy, the accumulation of nanoparticles at tumor sites is influenced not only by targeting ligands but also by systemic transport dynamics, vascular permeability, and immune interactions [175]. Research on polymeric and hybrid particulate systems demonstrates that optimizing geometry, mechanical properties, and hierarchical loading can significantly enhance therapeutic efficacy and retention in diseased tissue microenvironments [176]. In electrospray-generated PLGA systems, this tunability allows for the engineering of carriers with customized surface properties and internal architecture suitable for delivering cytotoxic drugs or immunomodulatory molecules, while maintaining structural stability and enabling controlled release.

Targeting strategies for electrospray-based PLGA nanoparticles in oncology and immunotherapy are generally categorized into passive and active approaches. Passive targeting takes advantage of physiological abnormalities, such as increased vascular permeability and impaired lymphatic drainage, to enhance nanoparticle accumulation in tumors [177]. In contrast, active targeting uses ligand–receptor interactions to improve selective cellular uptake, as shown with functionalized nanocarriers designed to interact with tumor-specific receptors [178]. These mechanisms often complement each other and are commonly combined in advanced nanomedicine platforms. The integration of passive accumulation and molecular recognition improves localization efficiency and enhances therapeutic performance. These principles are directly applicable to electrosprayed PLGA systems, whose tunable physicochemical properties significantly influence biodistribution and targeting behavior. The following sections will provide a comprehensive analysis of both passive and active targeting, with a focus on their application in electrospray-fabricated PLGA nanocarriers.

#### 6.2.1. Passive Targeting

Passive targeting through the enhanced permeability and retention (EPR) effect is a widely used mechanism for the accumulation of nanoparticles in tumor tissues. This principle is essential for designing electrospray-fabricated PLGA nanocarriers. The EPR effect results from various structural and functional abnormalities in tumor blood vessels, such as disorganized endothelial junctions, increased vessel fenestration, and inadequate lymphatic drainage. These conditions enable nanoscale carriers to escape circulation and enter the tumor interstitial space, where they can remain for extended periods. Consequently, these pathophysiological features create an opportunity for nanocarriers with optimized size and surface properties to preferentially accumulate in tumor microenvironments compared to normal tissues [179,180].

Electrospray processing is highly effective for engineering PLGA nanoparticles within the optimal size range for EPR-mediated aggregation (typically between 50 and 200 nm) and achieving narrow size distributions crucial for passive targeting efficiency. This method allows precise control over particle size, morphology, and polymer architecture by adjusting key parameters such as polymer concentration, solvent composition, voltage, and flow rate. These adjustments directly affect circulation kinetics and biodistribution profiles. While many passive-targeting studies have traditionally used conventional fabrication methods like emulsion–solvent evaporation, electrosprayed nanoparticles with controlled size and surface properties are increasingly recognized for their ability to achieve similar or improved passive accumulation. Additionally, they benefit from the monodispersity and reproducibility associated with electrohydrodynamic atomization [181,182]. Recent research by You et al. highlights the clinical advancement of these systems, which used electrospray-adjacent techniques to develop PLGA-based hybrid nanoparticles for ovarian cancer treatment. By achieving a uniform size of approximately 130 nm, these carriers effectively enhanced the EPR effect, allowing encapsulated PTX to reach therapeutic concentrations within the tumor while significantly reducing systemic side effects [151].

Preclinical studies show that PLGA-based nanocarriers use the EPR effect to enhance the tumor localization of various payloads, including chemotherapeutics and immunomodulators, compared to free drugs. For example, PEG-modified PLGA nanoparticles delivered systemically exhibit prolonged circulation and increased tumor accumulation via EPR, resulting in improved antitumor efficacy and reduced off-target toxicity in animal models [183]. While many studies focus on conventional PLGA systems, the mechanistic principles apply directly to electrospray formulations. These formulations can be designed to achieve similar passive targeting performance with potentially greater control over size and surface characteristics. A notable example is the work by Chatterjee et al., who synthesized ultra-small (~40 nm) electrosprayed PLGA nanoparticles for delivering methotrexate [183]. By exploiting the precision of the electrospray process, they developed smart monodispersed carriers that showed significant internalization in drug-resistant breast cancer cells through passive accumulation and micropinocytosis.

Despite its foundational role, the EPR effect exhibits significant inter- and intra-tumor heterogeneity, limiting its clinical reliability as a sole targeting mechanism [184,185]. Variations in vascular permeability, interstitial fluid pressure (IFP), stromal density, and perfusion result in inconsistent nanoparticle accumulation across different tumor types and among individual patients. These biological barriers hinder deep penetration of nanocarriers and can lead to uneven distribution within tumor masses, reducing therapeutic efficacy when relying solely on EPR. Consequently, research has increasingly focused on strategies to enhance EPR, such as co-administration with vascular modulation agents, using size-optimized carriers, or combining passive targeting with active targeting ligands to improve uptake and retention [186]. Recent studies suggest that active trans-endothelial transport mechanisms may contribute to the accumulation of nanoparticles in specific tumor environments, challenging the notion that gap-mediated extravasation is the sole process involved. While passive leakiness remains a significant factor in many cases, evidence indicates a more complex relationship between physical transport barriers and endothelial transcytosis, especially for nanoparticles that avoid rapid immune clearance and persist in circulation for extended periods [186].

For electrospray-based PLGA nanoparticles, precise control over size and surface properties can effectively navigate complex biological barriers. Optimizing particle dimensions and surface hydrophilicity, such as PEGylation, enhances systemic stability, prolongs circulation time, and increases tumor exposure [185,187]. Rational physicochemical tuning of nanocarriers is widely recognized as a key factor in biodistribution and therapeutic delivery efficiency in vivo [188]. Extended circulation increases the chances of repeated vascular passage and EPR-mediated accumulation. Additionally, combining passive targeting with active ligands or stimuli-responsive features has become a key strategy for addressing tumor heterogeneity and improving transport limitations [188].

Case studies demonstrating these principles include electrospray-derived carriers for chemotherapeutics like DOX and PTX. Tailoring the size and modifying the surface of these carriers enhanced tumor accumulation and improved antitumor activity in preclinical models. However, there are fewer direct reports on electrospray methods compared to traditional fabrication techniques. The potential for passive targeting in electrospray systems is further supported by studies involving PEG-PLGA nanoparticles, where controlling size and extending circulation time directly correlated with increased tumor deposition and therapeutic benefits in oncology models [148,182]. Furthermore, Kirtane et al. [189] demonstrated that reformulating toxic chemotherapeutics like tylocrebrine into size-optimized PLGA nanoparticles allowed the drug to utilize the EPR effect for targeted tumor localization. This method also prevented drug accumulation in the central nervous system, effectively addressing previous clinical challenges related to neurotoxicity. Innovation in this field is exemplified by the development of multi-layered particles. For instance, coaxial electrospray has been used to create core–shell PLGA structures that allow for the sequential release of PTX alongside secondary agents such as imatinib or DOX [111].

In immunotherapy, passive targeting enhances the delivery of immune modulators by increasing their local concentration in tumor microenvironments, which can improve antigen presentation and immune activation. Furtmann et al. [102] demonstrated the effectiveness of electrospray in producing 200 nm PLGA nanoparticles for delivering sensitive CMV peptides. The study showed that the gentle electrospray process preserved peptide bioactivity, achieving EE of 85% and resulting in stronger T-cell activation compared to larger microparticles. However, the immunosuppressive nature of many tumors, marked by high interstitial pressure and dense stroma, often requires additional active or stimuli-responsive mechanisms to achieve significant immunomodulatory effects [8,176]. Levy et al. (2021) demonstrated that electrosprayed PLGA nanoparticles co-encapsulating TLR9 and STING agonists significantly reduce tumor burden in metastatic models by maintaining high local concentrations of these agonists at the tumor site [190]. Recent studies on PLGA-based immunotherapies for colorectal cancer highlight that electrospraying provides the scalability and reproducibility needed for clinical applications. By precisely engineering nanoparticles to target the suppressive myeloid networks in the tumor microenvironment, these electrosprayed systems enhance the effectiveness of checkpoint blockades. This underscores the importance of combining passive targeting with tailored surface engineering and functional ligands [10].

#### 6.2.2. Active Targeting

Active targeting is a strategy that complements passive accumulation by using receptor-mediated recognition to improve cellular internalization and overcome physiological transport barriers that restrict nanoparticle uptake in tumors [10,118]. While passive targeting relies on systemic circulation time and size-dependent accumulation, active targeting modifies nanoparticle surfaces with ligands that specifically bind to receptors overexpressed on malignant cells, tumor vasculature, or immune subsets within the tumor microenvironment. These ligands can include antibodies, peptides, aptamers, polysaccharides, and small molecules, enabling receptor-mediated endocytosis and enhancing intracellular drug delivery [177]. Electrospray and electrohydrodynamic atomization techniques offer significant advantages for fabricating actively targeted drug carriers. Unlike emulsification methods that expose biomolecules to shear stress and phase interfaces, electrospray processing allows for solvent evaporation under milder conditions. This technique provides precise control over particle architecture and the distribution of functional ligands. It enables the incorporation of ligand-modified copolymers, the creation of core–shell structures through coaxial spraying, and the use of post-processing conjugation strategies. As a result, the density and orientation of ligands can be adjusted with greater reproducibility, which is essential for effective receptor binding. Experimental demonstrations of electrosprayed drug carriers have shown enhanced particle uniformity and improved encapsulation control compared to traditional fabrication methods, underscoring the technique’s relevance for functional targeting applications [191]. In electrospray, various validated strategies have been used to incorporate active-targeting ligands into polymeric nanoparticles while maintaining structural integrity and bioactivity. One common method involves blending ligand-bearing or functionally modified copolymers directly into the electrospray feed solution, enabling single-step assembly of surface-functional nanoparticles; for example, PLA–PEG copolymers processed by electrospray have been shown to retain functional surface moieties amenable to subsequent targeting [192]. A second route utilizes coaxial electrospray shell deposition, wherein the outer jet delivers ligand-rich polymer solutions that form the particle shell around a core payload, allowing independent control of targeting ligand presentation and drug encapsulation, as demonstrated in core–shell nanoparticles fabricated via coaxial electrospray with tunable outer compositions [15]. A third validated strategy involves post-fabrication conjugation: reactive functional groups are incorporated during electrospray processing and subsequently used for covalent attachment of targeting ligands under mild conditions, an approach successfully employed to tether thiolated peptides to electrosprayed-generated PLGA carriers [193]. These methods illustrate how electrospray affords structural precision and modular control over ligand presentation, supporting reproducible active targeting architectures in cancer therapy and immunotherapy platforms without compromising encapsulated payload stability.

Ligand-mediated targeting performed using PLGA nanoparticles has shown strong efficacy in enhancing chemotherapeutic selectivity. Folate receptor targeting remains one of the most investigated systems due to receptor overexpression in epithelial cancers. Experimental work on ligand-modified PLGA nanocarriers consistently reports significantly increased uptake and cytotoxicity in receptor-positive cell models compared with non-targeted controls, highlighting the impact of receptor-mediated endocytosis on therapeutic efficiency [10]. Similarly, transferrin-functionalized nanoparticles have demonstrated enhanced transport across cellular barriers, including the blood–brain barrier, owing to receptor-mediated transcytosis mechanisms. Evidence from targeted nanoparticle delivery studies indicates that exploiting endogenous nutrient transport pathways can improve brain tumor drug accumulation and therapeutic efficacy [10]. Hyaluronic-acid functionalization targeting CD44 receptors has also been widely investigated, particularly for drug-resistant tumor subpopulations and cancer stem cells. Targeted PLGA systems using this ligand exhibit improved retention and cellular uptake in CD44-expressing tumors, demonstrating potential to overcome chemoresistance mechanisms [194]. Although many of these demonstrations employ nanoprecipitation or emulsion fabrication, the physicochemical targeting principles translate directly to electrospray systems. Moreover, electrospray fabrication offers superior control over particle morphology and ligand distribution, suggesting potential for enhanced targeting fidelity.

## 7. Challenges and Future Perspectives

Despite the significant progress in electrospray-fabricated PLGA nanoparticles for drug delivery, several critical challenges remain that must be addressed to facilitate their broader clinical and industrial translation. One of the primary limitations is the scalability and reproducibility of the electrospray process. While electrospray enables precise control over particle size, morphology, and internal architecture at the laboratory scale, translating these capabilities to large-scale manufacturing remains challenging [12]. Issues such as jet instability, nozzle clogging, and sensitivity to environmental conditions can lead to batch-to-batch variability, which is unacceptable for pharmaceutical production. Advances in multi-nozzle systems, process standardization, and real-time monitoring strategies are required to improve throughput and ensure consistent product quality.

An additional but often overlooked challenge in electrospray-based nanoparticle production is the efficient collection and recovery of generated particles. During electrospraying, nanoparticles are typically deposited onto grounded collectors such as metallic plates, rotating drums, or liquid baths. However, particle losses due to adhesion to collection surfaces, dispersion in the surrounding environment, or incomplete transfer can significantly reduce overall process yield [81].

Collection strategies differ markedly in their recovery efficiency and economic feasibility. Solid collectors (e.g., metallic substrates) are simple and low-cost but often suffer from strong particle adhesion, requiring post-processing steps that reduce yield and reproducibility [118,195]. Liquid collectors can improve recovery efficiency by minimizing particle adhesion and facilitating direct suspension formation, although they introduce additional solvent handling and downstream processing requirements [196]. Cyclone collectors and electrostatic precipitators offer higher recovery efficiencies at larger scales by capturing airborne particles through inertial or electrostatic forces, making them more suitable for continuous processing; however, these systems involve higher equipment costs and operational complexity. Reported collection efficiencies vary widely depending on system design and operating conditions, ranging from moderate recovery in simple plate collectors to substantially higher yields in optimized cyclone or liquid-based systems. Efficient recovery is particularly important for formulations involving high-value therapeutics, where material loss directly impacts economic feasibility and scalability. Therefore, the development of integrated, scalable collection systems that balance recovery efficiency, cost, and process simplicity remains a key requirement for industrial translation of electrospray-fabricated PLGA nanoparticles [197,198].

Another important consideration involves regulatory challenges associated with PLGA-based nanomedicines. Although PLGA is an FDA-approved biodegradable polymer, the regulatory pathway for nanoparticle-based drug delivery systems remains complex. Factors such as particle size distribution, surface properties, drug loading efficiency, and release kinetics must be rigorously controlled and validated [11]. In addition, the lack of standardized characterization protocols and long-term safety data for nanocarriers continues to complicate regulatory approval processes. Emerging ML approaches offer promising tools to address these challenges by enabling data-driven prediction of nanoparticle properties and accelerating process optimization, thereby reducing reliance on empirical trial-and-error experimentation [191]. Furthermore, ML approaches can assist in modeling complex electrospray dynamics, drug release behavior, and even in vivo performance, enabling more efficient and rational design of delivery systems. Looking forward, future research should focus on advancing electrospray fabrication strategies and nanomedicine applications. Their ability to encapsulate and co-deliver multiple therapeutics enables the design of treatment strategies tailored to individual patient characteristics, including genetic profile, disease state, and drug response. This is particularly relevant for dual drug delivery systems, where synchronized and controlled release of multiple agents is re-quired. In addition to therapeutic applications, electrosprayed nanoparticles also show strong potential in diagnostic and theranostic applications. By incorporating contrast agents, imaging probes, or biosensors, these systems can enable targeted delivery while allowing the real-time monitoring of drug distribution, therapeutic efficacy, and disease progression. The development of multifunctional nanoparticles, achieved through surface modification and incorporation of diverse functional groups, further expands their capabilities for targeted delivery and controlled drug release. Future research should focus on advancing electrospray fabrication strategies and nanomedicine applications. This includes the development of scalable electrospray platforms, improved encapsulation techniques for sensitive biomolecules, and the design of multifunctional nanoparticles capable of targeted and stimuli-responsive drug delivery. In addition, integrating experimental studies with multiphysics modeling and data-driven approaches will be essential for achieving predictive control over nanoparticle performance. Another direction lies in the rational engineering of polymer chemistry and particle architecture to achieve programmable drug release profiles. By tailoring polymer composition, molecular weight, copolymer ratio, and end-group functionality, it is possible to modulate degradation behavior and drug–polymer interactions. In addition, controlling particle structure, such as internal morphology, multilayer configurations, or core–shell architectures, can further regulate diffusion pathways and release kinetics. These strategies enable the design of delivery systems capable of sustained, sequential, or pulsatile drug release, moving beyond conventional diffusion-controlled mechanisms. Such approaches are particularly relevant for advanced nanomedicine applications. In addition, integrating experimental studies with multiphysics modeling and data-driven approaches will be essential for achieving predictive control over nanoparticle performance.

Beyond conventional drug delivery applications, electrospray and related fabrication techniques have also been explored for emerging applications in cell-based therapies. In such systems, maintaining cell viability, functionality, and microenvironmental support during processing represents a significant challenge. Compared to scaffold-based approaches such as electrospinning and additive manufacturing, which provide structural frameworks for cell attachment and growth, electrospray-based methods remain less established for direct cell delivery due to potential stresses associated with high voltage, shear forces, and solvent exposure. Nevertheless, recent studies suggest that with appropriate process optimization, electrospray may offer opportunities for cell encapsulation and microcarrier fabrication, representing a potential direction for future research [199,200]. Overall, addressing these challenges through interdisciplinary efforts will be critical for translating electrospray-based PLGA nanoparticles from laboratory research to clinically viable and industrially scalable drug delivery systems.

## Figures and Tables

**Table 1:** A comparative overview of different nanoparticle fabrication methods.

Method	Principle	Process Complexity	Use of Surfactants	Particle Size Control	EE%	Suitability for Sensitive Biomolecules	Scalability/Yield	Key Limitations
Electrospray	Electrostatic forces generate charged droplets that solidify into particles	Moderate (requires voltage control but fewer steps)	Minimal or none	Excellent (monodisperse, tunable via parameters)	High (especially for hydrophobic drugs)	High (mild conditions, low shear)	Moderate–High (good collection efficiency)	Equipment sensitivity, lower throughput than spray drying
Emulsion–Solvent Evaporation	Formation of emulsion followed by solvent evaporation	High (multiple steps)	Required	Moderate (depends on emulsification energy)	Moderate	Limited (exposure to interfaces and shear)	Low–Moderate	Residual solvents, broad size distribution, surfactant residues
Nanoprecipitation	Polymer precipitation upon solvent–nonsolvent mixing	Low (simple mixing)	Often not required	Moderate	Moderate–High (for hydrophobic drugs)	Moderate (mild but solvent exposure)	Moderate	Limited for hydrophilic drugs, less structural control
Solvent Diffusion	Controlled diffusion of solvent into aqueous phase causing precipitation	Moderate	Often required	Moderate	Moderate	Moderate	Moderate	Process sensitivity, solvent residues
Spray Drying	Atomization of solution into hot gas leading to rapid drying	Moderate	Not required	Moderate	Moderate–High	Low–Moderate (thermal stress possible)	Very High (90–99%)	Thermal degradation, larger particle size range
Layer-by-Layer Assembly (LbL)	Sequential adsorption of oppositely charged layers	High (multi-step assembly)	Not required	Excellent (nanoscale precision)	High (for surface loading)	High (gentle conditions)	Low	Time-consuming, difficult scale-up

**Table 2:** Summary of electrosprayed PLGA nanoparticles encapsulating single or dual therapeutic agents, along with their fabrication parameters and formulation strategies for the treatment of various diseases.

Polymer	Solvent	Drug/Active Agent	Type of Particle	Particle Size	EE (%)	Disease	Experimental Model	Reference
PLGA (49:51) & mPEG-amine	Dichloromethane (DCM)	Curcumin	Single	<70 nm	~91%	Cancer	-	[91]
PLGA (85:15)	Dimethylformamide (DMF)/chloroform (1:3 *v*/*v*)	Curcumin	Single	122 ± 2 nm	Not-reported	Cancer	-	[92]
PLGA (85:15)	Chloroform: DMF = 7:3 (*v*/*v*)	Fe_3_O_4_, Netrin 1	Single	~3.0 μm	40.47 ± 5.30%	Cancer	-	[93]
PLGA & Hydroxyapatite	Not-reported	Pac-525	Single	Not-reported	Not-reported	Antimicrobial implant coating	-	[94]
PLGA (Resomer^®^ RG503)	DCM	SN-38 and Temozolomide	Single	1.58 ± 0.54 µm	Not-reported	Cancer	Rat cortical neurons/C57BL-6 J mice	[95]
PLGA (Resomer^®^ RG 503)	Hexafluoroisopropanol (HFIP)	Doxorubicin (DOX)	Single	6.74 ± 1.01 μm	Not-reported	Cancer	-	[96]
PLGA (50:50), Mw: 24,000–38,000 g/mol	Acetone	Thioguanine	Single	60 nm	~97.22%	Cancer	MC3T3-E1 cells	[97]
PLGA (Mw: 45,000 g/mol)	DMF	JNK inhibitor (IQ-1)	Single	0.9–1.3 μm	25–64%	Cancer	-	[98]
PLGA, mal-PEG-PLGA, CD44-PEG-PLGA	Acetone/DMF	Cisplatin	Single	550 ± 137 nm	Not-reported	Cancer	Sprague Dawley rats	[99]
PLGA & Sodium Alginate	DCM	Paclitaxel	Single	11.76 ± 2.79 μm	82 ± 0.1%	Cancer	-	[100]
PLGA (50:50)	DCM	Tacrolimus	Single	3–6 μm	84–91%	Immunosuppression /Transplantation	Sprague Dawley rats	[101]
PLGA (50:50, 7–17 kDa)	Dimethyl sulfoxide (DMSO): 2,2,2-Trifluoroethanol (TFE) (44:56 *v*:*v*)	Cytomegalovirus (CMV) and antigen peptides (pp65, IE-1)	Single	~200 nm	~85%	Immunotherapy (Antigen delivery, CD8^+^ T-cell activation)	-	[102]
Core: aqueous peptide solutions, Shell: PLGA (85:15)	Chloroform, Ethanol	Teriparatide and Hydrophobic Drug	Core–Shell	3.5 ± 0.5 μm	79.2 ± 19.8%	Cancer	HeLa cells	[103]
Core: DOX, Shell: PLGA	N,N-Dimethylacetamide (DMAC), Water	DOX	Core–Shell	189 nm	>83%	Cancer	-	[104]
Core: PLGA, Shell: Calcium Alginate	DCM, (core), Deionized water (Shell)	Paclitaxel (PTX)	Core–Shell	190.763 μm	78.31 ± 10.16%	Cancer	Human monocyte-macrophage MonoMac-6 cells	[105]
Shell: Eudragit/Core: PLGA	DCM, Ethanol/Water	Capecitabine and Osimertinib	Core–Shell	1.87 ± 0.23 µm	92.9% & 93.1%	Cancer	-	[106]
Shell: sodium hyaluronate, Core: IMN-loaded PLGA	DCM (core), Ethanol/Water, (shell)	PTX and Imatinib	Core–Shell	14.65 μm (avg)	94.4% & 97.5%	Cancer	CP70 and SKOV-3 cells	[107]
Core: PLGA Shell: PLGA	HFIP (core), DCM (shell)	PTX and Etoposide	Core–Shell	3.03 ± 0.5–3.04 ± 0.5 µm	85.8 ± 0.35%	Cancer	-	[108]
Core: PVP (containing CA4) or PCL (containing CA4), Shell: PLGA (containing DOX)	THF/Acetonitrile (ACN) (shell), ACN or Ethanol/DMF (core)	DOX and Combretastatin A4	Core–Shell	424.4 ± 78.9 nm for PVP-DOX/PLGA-CA4 and 455.7 ± 103.3 nm for PCL-DOX/PLGA-CA4	>90%	Cancer	Rat C6 glioma cells/BALB/c nude male mice	[15]
Shell: PLLA, Core: PLGA	DCM (core) DCM (shell)	Cisplatin	Core–Shell	0.47–5.31 μm	34–100%	Cancer	-	[109]
Core: Human Immunoglobulin G (IgG-FITC), shell: PLGA	Ethanol (shell), aqueous buffer (core)	Human Immunoglobulin-G (IgG-FITC)	Core–Shell	6.64 ± 4.55 µm	0.04–1.52%	Local immunotherapy/Sustained antibody delivery	INS-1 cells	[110]
Core: cisplatin Shell: PLGA	Aqueous solution water or buffer, (Core), DCM or chloroform (Shell)	Cisplatin	Core–Shell	850 ± 200 nm	(>70%)	Cancer	-	[14]
Shell: PLGA Core: PCL	Tetrahydrofuran and ACN (2:8, *v*/*v*) (shell), ACN(core)	Rhodamine B and Naproxen	Core–Shell	PCL-R/PLGA-N: 665.2 ± 45.9 nm, PCL-N/PLGA-R: 623.3 ± 53.2 nm	(>85%)	Cancer	Peripheral blood mononuclear cells	[111]
Shell: PLGA Core: PVP	Tetrahydrofuran and ACN (2:8, *v*/*v*) (shell), DMF–ethanol = 1:2 (core)	Rhodamine B and Naproxen	Core–Shell	PVP-R/PLGA-N: 404.2 ± 32.7 PVP-N/PLGA-R: 419.2 ± 23.5	(>85%)	Cancer	-	[111]
Outer layer: PEG, Middle layer: PLGA, inner layer: air	DCM (middle layer), Ethanol: water (9:1) (outer layer)	PTX and Osimertinib	Erythrocyte multi core–shell	846.9 ± 65.72 nm	~80%	Cancer	HEK293 cell line	[112]
Core: PCL-PEG Shell: PLGA	DCM	Resveratrol and Fibronectin	Core–Shell	1.30 ± 0.15 μm	78.5 ± 3.2%	Respiratory disease/Anti-inflammatory	-	[113]
Core: Chitosan, shell: PLGA (50:50) M_w_: 50,000 g/mol	1% Acetic acid, Acetone	Natamycin and Clotrimazole	Core–Shell	406.63 ± 8.37 nm	85.63 ± 0.04%	Antifungal therapy	MCF-7 human breast cancer cells	[114]
Core and shell: PLGA	DMAc: Acetone (3:7 *w*/*w*)	Saxagliptin, Dapagliflozin	Core–Shell	535 to 709 nm	EE >90%	Type 2 diabetes	-	[115]

**Table 3:** Drug release characteristics and therapeutic outcomes of PLGA-based systems.

Drug	Fabrication Method	Particle Size (nm)	EE (%)	Release Profile	Model (In Vitro/In Vivo)	Therapeutic Outcomes	Reference
DOX	Electrospray (coaxial, core–shell)	100–250	>80	Sustained release ~69% over 144 h	MCF-7 cells (in vitro)	Lower IC_50_ compared to free DOX; enhanced cytotoxicity	[104]
PTX	PLGA emulsion with cyclodextrin modification	148–164	60–86	Slower release, improved tumor accumulation	Mouse/rat (in vivo)	Prolonged circulation & enhanced tumor accumulation vs. Taxol^®^	[152]
PTX	Solvent evaporation (FA-PEG-PLGA modified)	120–150	70–80	Sustained release, GSH-responsive	Lung cancer xenografts (in vivo)	Higher tumor uptake & apoptosis; superior tumor growth inhibition vs. free PTX	[154]
DOX	Nanoprecipitation, PEGylated	110	95	Sustained release >80% over a period of 8 days	PC3, HeLa (in vitro)	Enhanced intracellular uptake & cytotoxicity	[155]
PTX (with imatinib)	Coaxial electrospray (core–shell microparticles)	micrometer scale (~14 μm)	-	Sequential release	Cervical cancer (in vitro/in vivo)	Therapeutic effect via staged drug release	[107]
PTX (chemo-photothermal)	Simple emulsion + Au half-shell + RGD	~130–150	75–95	Initial ~20%in the first 24 h then sustained up to ~92%	HeLa, MDA-MB-231 (in vitro)	Significant reduction in viability with NIR	[156]
PTX & DOX	Modified PLGA nanoprecipitation (not electrospray)	~94–133	-	-	GBM cells & rats (in vitro/in vivo)	Combination PLGA NPs showed improved tumor suppression and increased survival vs. free drugs	[157]
PTX + Curcumin-loaded PLGA	Nanoprecipitation	~<200	~6.3–8.9	Minimal ~72 h	MDA-MB-231 (in vitro)	Enhanced cytotoxicity vs. free drugs	[158]
Cisplatin	Electrospray (EHDA, coaxial & single needle	~100–200	>70	Controlled, slower release (core–shell vs. matrix)	Human ovarian carcinoma cell line (A2780) and its cisplatin resistant variant (A2780cis) (in vitro	A better response was obtained in both cell lines, lowering viability to 11% (A2780) and 51% (A2780cis) vs. 27% and 82% for free cisplatin	[159]
5-fluoroura-cil (5Fu)	Functionalized PLGA NP (EGF-targeted)	~200	~7	pH-responsive release	Human colorectal cancer cell line SW620 (in vitro) & mice (in vivo)	Higher tumor inhibition vs. free drug and non-targeted nanoparticles	[160]
Gemcitabine	Double emulsion solvent evaporation method	~100–200	~35	Sustained release	MIA PaCa-2 cell line (human pancreatic carcinoma) (in vitro)	Improved stability and tumor targeting	[161]
Cisplatin	Electrospray (EHDA, single needle)	~200–600	>70	Initial burst (<4 h) then sustained release	In vitro	Controlled release, characterized by tunable kinetics and morphology-dependent behavior, enabling optimized chemotherapy dosage	[162]
Cisplatin	Coaxial electrospray (core–shell EHDA)	~500	>70	More sustained vs. matrix nanoparticles	Human head and neck squamous carcinoma cell line (UM-SCC-47) (in vitro)	Improved cytotoxicity (EC50 = 6.2 µM) vs. free drug (9 µM) and uniform nanoparticles (7.6 µM), showing higher therapeutic efficacy via structural control	[14]
Carboplatin	PEG-PLGA nanoparticle system (using a double emulsion solvent evaporation method)	~100–200	~65	Controlled release	MSC-mediated delivery (in vitro)	Improved biocompatibility and delivery efficiency	[163]
Cisplatin (RGD-PLGA)	Double emulsion (surface-functionalized)	~150–200	~70–80	Sustained release	Lung cancer (in vitro/in vivo)	Enhanced therapeutic efficacy and safety, with improved pharmacokinetics and reduced systemic toxicity compared to free cisplatin	[164]

## Data Availability

No new data were created or analyzed in this study. Data sharing is not applicable to this article.

## Abbreviations

The following abbreviations are used in this manuscript:

PLGA	Poly(lactic-*co*-glycolic acid)
PGA	Polyglycolic acid
PLA	Polylactic acid
PCL	Polycaprolactone
Tg	Glass transition temperature
BSA	Bovine serum album
DCM	Dichloromethane
DMF	Dimethylformamide
HFIP	Hexafluoroisopropanol
DMSO	Dimethyl sulfoxide
TFE	2,2,2-Trifluoroethanol
DMAC	N,N-Dimethylacetamide
CMV	Cytomegalovirus
FEM	Finite element modeling
ML	Machine learning
CFD	Computational Fluid Dynamic
VOF	Volume of Fluid
DOX	Doxorubicin
PTX	Paclitaxel
